# Effectiveness of organization-directed interventions on healthcare professionals' well-being: a systematic review

**DOI:** 10.1016/j.eclinm.2025.103496

**Published:** 2025-09-13

**Authors:** Amber Boskma, Kim van der Braak, Lotty Hooft, Michiel Oerbekke, Arie Franx, Maarten van der Laan

**Affiliations:** a Department of Surgery, University Medical Center Groningen, the Netherlands; b Cochrane Netherlands, Julius Center for Health Sciences and Primary Care, University Medical Center Utrecht, the Netherlands; c Department of Obstetrics and Gynecology, Erasmus University Medical Center, Rotterdam, the Netherlands

**Keywords:** Healthcare professionals, Well-being at work, Work environment, Organization-directed interventions

## Abstract

**Background:**

Healthcare professionals' well-being at work seriously influences individuals, organizations, and care quality and safety. Many institutions focus on individual-level approaches. This review evaluates organization-directed interventions targeting the work environment of HCPs, promoting sustainable organizational conditions that support employee well-being and performance over time. Working conditions are considered part of the evidence-based protective factors for well-being at work, aligning with the approach of addressing underlying determinants rather than solely treating individual HCPs. This review aims to assess the effects of organization-directed interventions on hospital staff well-being, work environment, retention, and quality of care.

**Methods:**

MEDLINE, Embase, and CINAHL were searched from 2012 to Augustus 2025. Extracted data encompassed study characteristics, population details, intervention and comparator features, and outcomes. The Job Demands–Resources model was used as a theoretical basis. Risk of bias was evaluated. Interventions were categorized into *management & building, social resources & support, personal development & recovery, and multi-categorical*. Results were synthesized per outcome, effects were explored using forest plots, and the direction of effects is presented by Tables. Data analysis and visualization were performed using Excel and R. The protocol has been registered in May 2022 in PROSPERO (CRD42023390474).

**Findings:**

Screening of 3319 hits resulted in 54 included studies. Intervention effects are described and analyzed for outcomes: misconduct, workload, communication, job control, support, team climate, leadership, concentration, coping, efficiency, emotions, psychological characteristics, anxiety, burnout, depression, general health, job satisfaction, lack of energy, physical discomfort, quality of life, sleep, stress, employability, engagement, turnover intentions, patient quality, patient safety, and unwanted events. A total of 419 outcomes were extracted. Most studies showed some risk of bias, and the overall certainty of evidence was rated very low. The effect directions varied widely across outcomes, with standardized mean differences ranging from −3.28 to 3.56 and heterogeneous effects due to non-overlapping confidence intervals in most meta-analyses. Interventions focused on social resources & support, such as the rational emotional intervention, consistently yielded positive effects across different outcome domains. However, some management & building interventions, such as moderated solution meetings, were linked to worse outcomes like lower job satisfaction and quality of life.

**Interpretation:**

Findings should be interpreted with caution due to the overall low quality of the evidence. Furthermore, the overall findings are mixed. Some interventions demonstrated beneficial effects for certain outcomes while showing neutral or even detrimental effects for others. These variations underscore the importance of further research with more rigorous study designs, larger sample sizes, a consistent core outcome set, conducting a thorough needs assessment in advance, understanding potential contributors to reduced well-being, and considering local contextual factors before selecting or implementing interventions.

**Funding:**

10.13039/501100012524NFU and 10.13039/501100010763ZIN.

**Research in context**

**Evidence before this study**

The research on well-being of healthcare professionals (HCPs) has been growing rapidly the last decade with an exponential surge starting from the COVID pandemic. A large portion of the studies were therefore COVID-related. Additionally, a significant number of studies focused on evaluating a single intervention on a single outcome. Most studies seem to neglect that well-being is a multi-dimensional concept. However, previous reviews did not include a wide range of outcomes (e.g., focusing only on mental health). Moreover, some reviews aimed including workplace/organizational-directed interventions but included interventions on individual adjustments (e.g., skills and knowledge development). The collected body of knowledge is dispersed and focused on personal resilience. It does not take culture, system or context into account. The focus is rather on treating the individual HCP than on the underlying organizational determinants.

**Added value of this study**

This review is a comprehensive overview of organization-directed interventions to improve healthcare professionals' well-being. Within this study, well-being is operationalized using the Job-demands resources model which supports a complete perspective and a wide range of outcomes. Moreover, by including only studies after 2012, interventions were gathered relevant to current practice. Lastly, by avoiding interventions aiming at adjusting individuals and focusing solely on interventions targeting organizational and systemic changes, this review provides essential guidance for selecting and developing organization-directed interventions that are more symptom-controlled, sustainable, and contributing to a more supportive work environment.

**Implications of all the available evidence**

We identified 54 studies detailing organizational-directed interventions targeting healthcare professionals, including nurses, doctors, and other staff, focusing on their well-being, work environment, retention, and quality of care in hospitals. Interpreting the effects of these interventions reveals a mixed picture: some improve well-being, while others may be detrimental. Certain interventions are effective in one aspect of well-being but negatively impact another, complicating decision-making. Interventions emphasizing *social resources & support* showed the most consistent improvements across various outcomes. However, their effectiveness is uncertain due to large heterogeneity and small sample sizes. Healthcare organizations must carefully consider contextual factors, such as understanding the causes of outcomes, for effective decision-making. A one-size-fits-all approach is not feasible. To make better-informed decisions with greater certainty and less data heterogeneity, consensus is needed on the aspects of healthcare professionals' well-being and standardized measurements with larger sample sizes.

## Introduction

Improving employee health is top priority in hospital strategies,^1^^,^^2^ especially as healthcare systems face rising demand and shortages of professionals.^3^ Addressing healthcare professionals' (HCP) working conditions and well-being is crucial for safe, high-quality care.^4^, ^5^, ^6^, ^7^ Well-being at work involves ‘*creating an environment promoting contentment and allows employees achieving full potential, benefiting both themselves and their organization*’.^8^

Multi-level strategies are more effective than individual interventions for improving HCPs' well-being.^9^, ^10^, ^11^ Here, individual-directed interventions focus on physiological, emotional, or behavioral changes,^12^ while organization-directed interventions target systemic issues as policy and processes^12^ and impact larger groups of HCPs, are more symptom-controlled, sustainable, and integrate into organizational culture, ultimately boosting engagement and morale. Essentially, interventions can be distinguished based on whether they address persons (individual-directed interventions) or address contextual determinants (organization-directed interventions). Research shows that organization-directed interventions improve well-being, work engagement, and quality of life.^13^

However, many institutions focus on individual-directed interventions,^14^^,^^15^ which places responsibility on individuals and offers limited statistical evidence of effectiveness.^16^^,^^17^ Programs for peer-support and resilience,^18^^,^^19^ for example, are offered but provide weak evidence. Furthermore, the effect of such interventions may not always be beneficial for HCPs. For example, literature indicates that non-professionally organized (peer)support for resilience can have negative effects on well-being outcomes.^20^ Organization-directed interventions can address systemic causes of stress and dissatisfaction, similar to treating root cause of diseases rather than its symptoms.

Despite their complexity, including various working mechanisms within healthcare settings and resource demands, organization-directed interventions are necessary for sustainable improvements in HCPs' well-being. There is a need to provide an overview of organization-directed interventions and their (un)beneficial effects on multiple outcomes in different contexts. Understanding where the beneficial and unbeneficial effects on HCPs occur needs to be recognized when selecting, performing, and evaluating such an organization-directed intervention. A shift in focus of these efforts of symptom control is needed to tackle root causes.^21^ This study aims to provide an evidence-based overview of organization-directed interventions for healthcare professionals' well-being, work environment, retention, and quality of care in hospital settings. Nevertheless, working conditions are considered as protective factors for well-being at work.^22^ Applying insights from this overview of effective interventions that adjust contexts will help create a sustainable and supportive work environment for the future.^23^

## Methods

This systematic review and explorative meta-analysis are developed and reported according to PRISMA guidelines.^24^ The protocol has been registered in May 2022 in PROSPERO (CRD42023390474).^25^

### Search strategy and selection criteria

Studies that targeted HCP in hospitals, evaluated organization-directed interventions, compared these with no intervention or other organization-directed interventions, used experimental designs, focused on well-being, work environment, retention, or quality outcomes, and were published after 2012 for relevant interventions for daily practice^26^ in English were considered eligible. Studies targeting individual HCPs' psychological, emotional, or behavioral response or coping mechanisms were excluded. Studies were searched in Augustus 2025 in MEDLINE Ovid, Embase Ovid, and CINAHL using terms such as ‘healthcare professionals’, ‘hospital’, ‘well-being’, and ‘controlled before-after studies’ (see Additional File 1 for the complete search strings). Titles and abstracts were screened in pairs, after the first 10% discrepancies were resolved. Full-text screening followed with consensus on discrepancies.

### Data analysis

AB and KvdB extracted data using a standardized, piloted Excel form. Extracted data were^27^: study, population, intervention/control characteristics, and outcomes of interest. Personal communication with the WHO and developer of the Job-demands resources model (JD-R model)^28^ informed the construction of seven outcome categories: six JD-R categories and ‘Quality of care’. Risk of bias and GRADE was assessed by AB and checked by KvdB.^29^ Disagreements were discussed and solved. AB and KvdB categorized interventions inductively into four types and sub-categories: (1) *Management & Building* (workhours, continuous improvement, environment, equipment support & patient handling, workflow improvement, care model); (2) *Social resources & support* (emotional support, (additional) staff support, optimizing teams); (3) *Personal development & recovery* (role opportunities, relaxation opportunities, other team/setting opportunities); (4) *Multi-categorical* (interventions that contain elements from several of the previous categories, detailed in Additional File 2). Results per study per outcome domain were characterized by their effect direction (favorable intervention/control, unclear, no change) based on author reports.^30^^,^^31^ For multiple measurements in a single outcome domain, the overall effect was based on the majority. An effect was unclear when insufficient information was reported to characterize the direction. When studies reported mean differences of 0, the effect was indicated as no change. Feasibility of meta-analyses was assessed, and forest plots with standardized mean differences (SMDs) were created for continuous variables.^32^ Plots were created in R statistics 4.2.2 (Foundation for statistical computing, Austria) to explore and visualize effects. Due to heterogeneity, no pooled effect is presented. To account for heterogeneity, studies are presented within intervention categories, and as much information as possible is included in plots, such as intervention/comparator and measurement instrument. Records and data are managed using Endnote 20.1 (Clarivate Analytics, US), Rayyan (Rayyan systems Inc, US), Excel, and Mendeley Reference Manager 2.590 (Elsevier Inc, New York).

### Ethics

No human subjects were involved since this is a review of literature. This study does not require ethical approval. There are no ethical, legal or security issues regarding the data collection, processing, storage and dissemination in this project.

### Role of the funding source

This research was carried out within the health care worker resilience and well-being program of the Consortium Quality of Care of the Netherlands Federation of University Medical Centers (NFU). Additionally, the Dutch National Health Care Institute (ZIN) made funding available. The funders had no role in study design, data collection, data analysis, data interpretation, or writing of the manuscript.

## Results

**Fig. 1:**
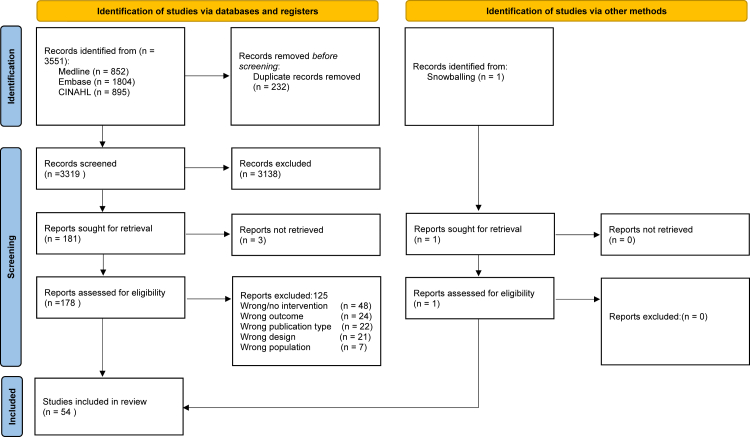
**Flow diagram.**^87^

The search retrieved 3319 unique records, with 54 studies included in this review^33^, ^34^, ^35^, ^36^, ^37^, ^38^, ^39^, ^40^, ^41^, ^42^, ^43^, ^44^, ^45^, ^46^, ^47^, ^48^, ^49^, ^50^, ^51^, ^52^, ^53^, ^54^, ^55^, ^56^, ^57^, ^58^, ^59^, ^60^, ^61^, ^62^, ^63^, ^64^, ^65^, ^66^, ^67^, ^68^, ^69^, ^70^, ^71^, ^72^, ^73^, ^74^, ^75^, ^76^, ^77^, ^78^, ^79^, ^80^, ^81^, ^82^, ^83^, ^84^, ^85^ (one added through cross-referring^86^). Fig. 1 and Table 1 present the study selection flow and characteristics, respectively. Participants were nurses, doctors, and/or other professionals. Sample sizes ranged from 4 to 9000, with one study reporting the number of measurements instead of the number of individuals.^65^ Intervention types (subcategories) included: (a) *Management & Building* (workhours, continuous improvement, environment, equipment support & patient handling, workflow improvement, and care model); (b) *Social resources & support* (emotional support, (additional) staff support, and team optimalization); (c) *Personal development & recovery* (role opportunities, relaxation opportunities, and other team/setting opportunities); and (d) *Multi-categorical*.

**Table 1:** Sample characteristics.

References	Design	Sample			Characteristics of the intervention and the comparator		Outcomes	Measurement instrument
Author Publication year Country		Healthcare professional Sample size: (N = )	Age: mean (SD)	Gender: % female				
Management & building | workhours								
Richter (2014)^61^ Germany	Before/after study	Physicians (N = before: 328, after: 994)	Before: 39.4, (9.1), After: 39.8 (9.3)	Before: 29.4% female, After: 36.6% female	European Union Working Time Directive.	No European Union Working Time Directive.	Sick Leave, burnout (emotional exhaustion, depersonalization, personal accomplishment)	Maslach burnout inventory, Sick leave from system
Ripp (2015)^64^ USA	Before/after study	Internal medicine physicians (N = before: 108, after: 123)	NR	Before: 56% female, After: 42% female	The July 2011 resident physician duty-hour regulations: 16-h continuous duty rule.	No duty hour restrictions.	Burnout prevalence, burnout, excessive sleepiness	Epworth sleepiness scale, Maslach burnout inventory
Turunen (2020)^72^ Finland	Quasi-experimental study	Nurses, midwifes, practical nurses, administrative assistants, pharmacists, physiotherapists, and other non-nursing professions (N = approximately 9000)	NR	89% female	Participatory working time scheduling software empowers hospital staff to help create schedules. It balances employee preferences with staffing needs, legal rules, and fairness. Staff submit preferred shifts within shift demands, while ward-level rules guide the process transparently.	Traditional scheduling. No participatory working time scheduling software.	Incident sickness absence spells, number of short sickness absence days per employee	Unknown instrument
Webster (2019)^75^ Australia	Before/after study	ICU nurses (N = before: 114, after: 152)	NR	79% female	8 1⁄2-h shifts, including 30-min meal break representing the standard working model for providing 24-h nursing care.	12-h shift rostering.	Satisfaction with roster, sleep, sick leave, number of incidents, accessed professional development, turnover	Self-devised, hospital administrative data on sick leave and incidents
Fond (2023)^84^ France	Quasi-experimental study	Nurses and nurse assistants (CG 1811; IG 1322)	40.88 (10.15)	99.86% female	12-h shifts.	7-h shifts.	Psychological demand, decisional latitude, social support, burnout	Job content questionnaire, Maslach burnout inventory
Fratissier (2021)^81^ France	Before/after study	ICU nurses and nursing auxiliaries (N = 116)	Age: <45 years: 79.3%; ≥45 years: 20.7%	NR	12-h shifts.	Two alternating day shifts (morning or afternoon) and one night shift in 10 h.	Time pressure difficulties, work-life balance, fatigue and/or weariness, sleep disorders attributed to work, upper limb complaints attributed to work, complaints concerning lumbar vertebrae attributed to work	EVREST questionnaire
Battle (2018)^35^ UK	Before/after study	ICU nurses (N = 123 (CG: 60, IG: 63))	NR	NR	12-h shifts.	8-h shifts.	Burnout (emotional exhaustion, depersonalization, personal accomplishment), sickness, personal injuries	Maslach burnout inventory, Number of physical injuries reported each month. All sickness data were taken from the official system
Levin (2019)^48^ Canada	Randomized cross-over trial	Physicians (N = 124 (CG: 60, IG: 64))	CG: 28.9 (3.7), IG: 30.1 (5.5)	CG: 47% female, IG: 52% female	Casino shifts either from 2000 to 0400 h (casino A) or 0400–1200 h (casino B). Attending physicians all worked standard shifts during the entire 12-month period.	Physicians worked standard overnight shifts from midnight to 0800 h.	Resident well-being, mood	Brief resident wellness profile
Lucas (2012)^52^ US	Randomized controlled trial	Physicians (N = 60)	Median: 38 (range 29–55)	48% female	Assignment to random sequences of 4-week rotations.	Assignment to random sequences of 2-week rotations.	Perceived stress, emotional exhaustion, inadequate workplace control, burnout	Minimizing error, maximizing outcomes, Maslach burnout inventory, National job burnout survey, Perceived stress scale
Lindeman (2013)^50^ USA	Before/after study	Physicians (N = before: 108, after: 104)	Mean: 30 (range 25–36)	NR	The July 2011 resident physician duty-hour regulations: 16-h continuous duty rule.	Before the implementation of duty hour regulation.	Quality of life (balance with personal commitments, balance with family, optimal functioning), satisfaction (work life quality, personal life quality, sleep amount), patient care quality, health, mental wellness, burnout, sleepiness	Center for epidemiologic studies depression scale, Epworth sleepiness scale, Maslach burnout scale, Self-devised, Short-form health survey
Nomura (2016)^54^ Japan	Before/after study	Pediatric physicians (N = before: 34, after: 41)	29.0 (1.9)	44% female	Duty hour regulations. An overnight call shift system was implemented in July 2011 to address the extended duty hours for pediatric residents.	No duty hour regulations.	Depression	Center for epidemiologic studies depression scale
Parshuram (2015)^58^ Canada	Randomized cross-over trial	ICU physicians (N = 41 (CG: 13, IG1: 14, IG2: 14) (807 patients))	Age 25–30: 66%, age 30–35: 21%, age >35: 9%, unknown/not reported: 4%	NR	Intervention 1 used 16-h overnight shifts (4:30 pm–8:30 am) with 24 h off. Weekdays had 8 am–5 pm shifts, and one resident worked weekends. Intervention 2 used 12-h overnight shifts (8:30 pm–8:30 am) for 3–4 nights, followed by 72 h off. Day shifts ran 8 am–4:30 pm or 8:30 pm, requiring an extra 8:30 pm handover.	24-h schedule (standard for Canadian ICU's): the resident's duty period began at 8 am and finished at 8:30 am the next morning. Overnight duty was followed by 24 h free of duty.	Sleepiness during the day, sleepiness during the night, burnout (depersonalization, emotional exhaustion, personal accomplishment), preventable adverse events, mortality	Maslach burnout inventory, Stanford sleepiness scale, Trained nurses observed handover, and multidisciplinary ward rounds
Rodriguez (2020)^65^ UK	Interrupted time series	Mental health ward nurses and patient care assistants (N = average number of staff per month before: 31.35, after: 29.21. Total number of observed weeks per ward before: 296, after: 167)	Before: 45.01 (4.12), after: 44.74 (4.05)	Before: 79.40% female, after: 78.03% female	12 h shifts.	8 h shifts.	Sickness absences	Hospital administrative data on sickness
Management & building | continuous improvement								
Uchiyama (2013)^73^ Japan	Randomized controlled trial	Nurses (N = 308 (CG: 163, IG: 145))	CG: 31.7 (9.1), IG: 33 (9.6)	CG: 97.6% female, IG: 100% female	Participatory intervention with a 3-month intensive period followed by 3 months of implementation. Subchief nurses acted as key facilitators, attending group meetings, sharing challenges, and receiving guidance. They relayed information to staff and tracked progress via task sheets. A booster session after two months reviewed unit activities.	No participatory intervention.	Mental health, job demands, job control, supervisor support, coworker support, effort, reward, goals, efficiency, participatory management, competence development, work climate, leadership, feedback	Job content questionnaire, Quality work competence questionnaire, Center for epidemiology studies depression scale
Luo (2022)^53^ China	Controlled before/after study	Obstetric nurse (N = CG: 51, IG: 146)	CG: 32.82 (6.5), IG: 34.53 (6.5)	CG: 100% female, IG: 99% female	Continuous plan-do-check-act cycle was implemented. Nurses in the experimental group were trained on the obstetric nursing-sensitive quality indicators online and offline based on the KAP model. The indicators were applied in clinical practice, and obstetric nursing quality was continuously improved.	The quality control group included 2 head nurses, 8 delivery room leaders, 3 ward leaders, and 6 primary nurses. Head nurses organized seminars on quality indicators, while other leaders collected data. Monthly meetings focused on quality improvement strategies.	Job satisfaction	Obstetric nurses' job satisfaction questionnaire
Pan (2022)^57^ China	Non-randomized controlled trial	Nurses (N = 160 (CG: 80, IG: 80))	CG: 31.56 (1.34), IG: 31.64 (1.33)	98% female	PDCA cycle management model to ensure continuous improvement. The model comprises four stages: Planning, Implementation, Inspection, and Summary and Treatment Stage.	Routine nursing management.	Job satisfaction (family/work balance, job recognition, personal growth and development, wages and benefits, the work itself, relationship with colleagues, workload, management)	Job satisfaction rating scale
Niks (2018)^55^ Netherlands	Quasi-experimental study	Nursing department, laboratories, emergency department (N = at T1: 60 nurses, CG: 32, IG: 28. N = at T1: 35 laboratory, CG: 18, IG: 17)	Nursing department CG: 34.1 (10.8), IG: 40.4 (10.2); laboratory department CG: 45.5 (10.5), IG: 48.6 (11.4)	Nursing department CG: 90.6% female, IG: 92% female; laboratory department CG: 73.7% female, IG: 88.2% female	The DISCovery method consisted of three successive steps: (1) psychosocial risk diagnosis; (2) development of interventions; and (3) implementation of the interventions.	No DISCovery method.	Cognitive resources, emotional resources, physical resources, cognitive detachment, emotional detachment, physical detachment, work satisfaction, individual work performance, work performance team, work break conditions, concentration problems, teamwork, work satisfaction	Unknown instrument, VAS, DISC questionnaire, One item: “I am satisfied with my present job”, Rating own work performance and the work performance of their team
Schneider (2019)^66^ Germany	Interrupted time series	Emergency department physicians, nurses and administrators (N = 41)	NR	NR	Ten multi-professional meetings in which emergency department physicians and nurses developed solutions to work stressors in a systematic moderated process. Most solutions were consecutively implemented.	Control period with no meetings developing stress solutions.	Work system factors (patient stressors, job control, participation opportunities, work overload, personnel resources, information problems, uncertainty, overtime, social support, supervisor feedback), provider well-being (emotional exhaustion, depersonalization, depressive symptoms, job satisfaction, turnover intentions), quality of care (frequency of errors, patient safety)	Systems engineering initiative for patient safety, Maslach burnout inventory, One-item job satisfaction, Patient health questionnaire, One-item turnover intention, 3-item scale on the frequency of medical errors, One- item “Please rate the degree of patient safety in your department from your point of view”
von Thiele Schwarz (2015)^74^ Sweden	Quasi-experimental study	Physicians, nurses, assistant nurses, physiotherapists, managers, medical secretaries (N = 195 (CG: 90, IG: 105))	CG: 45 (12.1), IG: 46.7 (9.2)	CG: 91.2%, IG: 96.4	Health protection through Kaizen involves regular unit meetings to identify, discuss, test, and evaluate work problems. One to three employees act as representatives. Meetings occur 1–4 times per month.	No continuous improvement system (Kaizen).	Workability, productivity, self-rated health, self-rated sickness (frequency, duration)	One-item general health, Health and work questionnaire, One-item work ability index, Self-rated sickness with STEM questionnaire
Mericle (2023)^82^ USA	Before/after study	Nurse managers (N = 14)	NR	NR	PDCA improvement plan developing and implementing a nursing leadership action plan regarding wellbeing through a focused improvement event.	No improvement initiative.	Distress, autonomy, growth and development, true collaboration, work-life balance, health and safety, reward and recognition, scope and responsibility/span of control, authentic leadership, culture of accountability	Subjective Units of Distress Scale
Management & building | environment								
Feeley (2019)^42^ Canada	Before/after study	NICU nurses (N = before: 54, after: 54)	32.94 (9.8)	96.3% female	The ward was redesigned with five 6-bed pods and 10 family rooms. New-borns start in pods for acute care and move to family rooms before discharge. Two rooms have full-size beds, and family rooms have lounge chairs for overnight stays. The unit features new equipment, indirect lighting, large windows with blinds, and portable phones for emergencies.	Previous design of the ward was an open ward with florescent lighting and windows on only 1 of the 4 walls. There was 1 room designated for mothers to express breast milk, and a parent room with 1 sofa bed for overnight stays.	Total stress, total obstacles, support from colleagues, support from supervisors, team effectiveness, global work satisfaction, adverse events	Team effectiveness tool, Job content questionnaire, Global measure of work satisfaction, Nurse stress scale, Data on incident reports
Bragard (2013)^37^ Belgium	Randomized cross-over trial	Physicians, nurses, psychologists, and secretaries of the radiation therapy department (N = 25)	36.6 (7.7)	84% female	The subjects used Luminette light glasses at work in the morning between 7:00 and 9:00 for a maximum of 30 min daily at least five days a week.	Not using Luminette.	Depression, sleepiness, general health, physical functioning, pain, emotional wellbeing/problems	Beck depression inventory, Epworth sleepiness scale, Short Form 36 survey
Copeland (2017)^40^ USA	Quasi-experimental and before/after study	Acute care unit nurses (N = before: 26, after: 35)	Before: 24.4 (9.8), after: 35.7 (10.7)	Before: 92% female, after: 91% female	Centralized nurses' stations.	Decentralized nurses' stations.	Job satisfaction, patient fall data	VAS, Electronic patient record data
McNeer (2016)^56^ USA	Randomized cross-over trial	Anesthesiologists (N = 20)	NR	40% female	Lunch breaks in noisy environments.	Quite lunch breaks.	Workload, lack of Energy, lack of motivation, physical exertion, physical discomfort, sleepiness	NASA task load index, Swedish occupational fatigue inventory
Simons (2018)^68^ Netherlands	Randomized cross-over trial	ICU nurses (N = 10)	34	70% female	High-intensity dynamic lighting provides up to 1700 lux (compared to 300 lux in standard settings) and adjusts colors based on a fixed rhythm, using ceiling-mounted fluorescent tubes in patient rooms.	Standard lighting settings.	Depression, fatigue, quality of life, sleep quality, feeling dull, feeling good, subjective sleep duration, activity, positive thoughts, positive events	Rating scale diary, Center for epidemiology studies depression scale, Fatigue assessment scale, World health organization quality of life scale
Tseng (2022)^71^ Taiwan	Non-randomized controlled trial	Operating room circulating nurses and nurse anesthetists (N = 20 circulating nurses, 16 nurse anesthetists, 18 operations with noise and 18 operations with popular Chinese songs, 17 operations with radio and 17 operations with Mozart)	NR	NR	Music during operations was tested. The experimental group received next to operating noise three types of music (surgeries with popular Chinese songs, FM radio talk show programs, and Mozart's music). The volume of music was also considered (55–60 dB vs. 75–80 dB, within-subjects).	Operating noise only	Workload, anxiety/emotional states	Subjective workload assessment technique questionnaire, State-trait anxiety inventory-state
Management & building | equipment support & patient handling								
Dennerlein (2017)^86^ USA	Controlled before/after study	Registered nurses, licensed practical nurses, clinical nurse specialists, and patient care assistants (N = before: hospital A: 580; hospital B: 1011, after: hospital A: 499; hospital B: 971)	Hospital A: 42.7 (0.49), hospital B: 40.6 (0.43)	Hospital A: 93.5% female, hospital B: 91.4% female	The safe patient handling and mobilization program. The program included an organizational policy, the investment in equipment, broad-based training, and risk assessments. Additionally, the program included building a hospital-wide infrastructure for maintaining and servicing equipment, providing clean slings, embedding the use of equipment and practices into the care plan for each patient, implementing a mentoring program and utilizing a strong communication program with leaders, workers and clients.	No safe patient handling and mobilization program.	Any pain, work interference, safe patient handling, unsafe patient handling, safety practices	Nordic occupational safety climate questionnaire, Question: In general, how much did this pain interfere with your normal work? Three questions asked how often workers 1) transferred patients who could not bear weight without the use of equipment but with the help of a coworker, 2) transferred patients who could not bear weight without the use of equipment or the help of a coworker, and 3) transferred patients who were combative patients
Risor (2017)^63^ Denmark	Controlled before/after study	Nurses, service assistants, and therapists (N = CG 2010: 201, CG 2011: 172, IG 2010: 293; IG 2011: 271)	CG 2010 (number, %) <24: 4 (2%), 25–34: 85 (42%), 35–44: 48 (24%), 45–54: 31 (15%), 55–70: 28 (14%), unknown: 5 (2%), CG 2011 (number, %) <24: 9 (5%), 25–34: 69 (40%), 35–44: 36 (21%), 45–54: 31 29 (17%), 55–70: 20 (12%), unknown: 9 (1%), IG 2010 (number, %) <24: 10 (3%), 25–34: 128 (44%), 35–44: 53 (18%), 45–54: 64 (22%), 55–70: 34 (12%), unknown: 4 (1%), IG 2011 (number, %) <24: 15 (6%), 25–34: 117 (43%), 35–44: 51 (19%), 45–54: 58 (21%), 55–70: 25 (9%), unknown: 5 (2%)	CG 2010: 95% female, CG 2011: 93% female, IG 2010: 92% female, IG 2011: 92% female	Patient-handling equipment intervention. (1) Developing and sharing patient-handling guidelines to clarify responsibilities among staff groups and ensure proper use of equipment; (2) Establishing guidelines for purchasing new equipment, with a focus on reducing high physical workloads like manual patient lifting; (3) Allocating funds to purchase new patient-handling equipment at a rate of V13,300 per bed-ward; (4) Implementing a comprehensive training program for nursing staff in intervention bed-wards, including local instructors, manager training, and full-day training for other staff. New nursing staff received two-day training, covering the use of patient-handling equipment; (5) Weekly visits from the project manager to provide support and guidance to local instructors, managers, and nursing staff as needed.	No intervention.	Lower back problems, lower back medical support, absent due to lower back problems, neck and shoulder problems, neck and shoulder medical support, absent due to neck and shoulder problems, knee problems, knee medical support, absent due to knee problems, hand wrist problems, hand wrist medical support, absent due to hand wrist problems, aggression (experienced physically aggressive episodes, average number of physically aggressive episodes, experienced mentally aggressive episodes, average number of mentally aggressive episodes), staff with good or better health, number of accidents, number of accidents low-back	Respondents were asked if they had experienced physically or mentally aggressive episodes within the last 12 months, and if so, how many episodes. Respondents were asked about how many days they had experienced pain or disorder within the last 12 months. Respondents were asked if they had experienced a work-related accident within the last 12 months. Short-form health survey
Li (2021)^49^ China	Controlled before/after study	Nurses (N = CG: 10, IG: 10)	NR	NR	Standardized exercise plan for total knee arthroplasty patients which was showed through a 3-min video and a bedside interactive system for playing the video.	Patients received oral education, exercise demonstrations via a bedside interactive system, and quizzes to assess their understanding of the exercise plan, with additional tutorials as necessary.	Burnout (emotional exhaustion, depersonalization, reduced personal accomplishment), job stress	Nurses job stressors scale, Maslach burnout scale
Davalos (2024)^85^ USA	Non-randomized cross-over trial	Orthopedic surgery interns and residents (N= CG: 23, IG: 17)	NR	NR	Automated electronic medical record inpatient tracking lists	Manual electronic medical record patient lists	Negative impact on sleep, affecting job satisfaction, negative impact on patient care, workload	Self-devised
Management & building | workflow improvement								
Thanarajasingam (2012)^70^ USA	Interrupted time series	Internal medicine physicians (N = 280. 15,926 patients on resident and nonresident services)	NR	NR	A census cap implementation, setting a maximum number of patients per department. A unit-based admission process grouped patients and care teams by department, with each physician overseeing a specific unit. This arrangement brought all healthcare professionals and patients together in one location.	No consensus limit and no unit-based admission process	Census/caseload was appropriate, opportunity to manage diverse pathology, opportunity and guidance to develop skills, patient safety (rapid response team events, cardiopulmonary resuscitation events, intensive care unit transfers, patient safety indicators, readmission within 30 days)	Self-devised, Unknown instrument
Hess (2015)^45^ Switzerland	Before/after study	Emergency department physicians, general practitioners, and nurses (N = before: 20, interim: 18, after: 22 participants)	36.3 (8.3)	84.2% female	A hospital-based primary care center was implemented with redesigned patient flow and integrated general practitioner services. GPs can work at the new center or the traditional out-of-hours service. Patients are triaged by a nurse and assigned to either the primary care center or emergency department based on severity.	Before the intervention, all emergency patients were treated in the traditional emergency department.	Overall job satisfaction	VAS
Management & building | care model								
Hansson (2020)^80^ Sweden	Before/after study	Midwives (N = before: 58, after: 58)	Mean 44 (range 27–65)	NR	Midwifery model of care. The model includes among other things: midwife is together with the woman, using grounded knowledge, forming a reciprocal relationship to create a birthing atmosphere, women are centered.	No midwifery model of care.	Social support, work ability (knowledge, mental, emotional, collaborate, physical), worrying about reorganization, worrying about technology, worrying about manage at work, worrying about get unemployed, worrying about bullying, worrying to sex harassment, climate (boss consider your opinions, conflict involvement, uneasiness going to work), stress Burnout (personal, work related, client-related), demand, control, work environment (engagement, high demands on oneself, hard to say no, responsibility), sense of coherence	5-item job demands scale, 6-item job control scale, Karasek scale, Work stress questionnaire, Copenhagen burnout inventory, Perceived stress scale, unknown instrument
Social resources & support | emotional support								
Liu (2022)^51^ China	Before/after study	Emergency department nurses (N = 50)	25.75 (2.64)	92% female	The rational emotional intervention included weekly 1-h sessions where nurses discussed their stress, anxiety, and nervousness in a calm setting. Emotional expressions were observed to identify causes of distress, while hierarchical management provided support and education.	No intervention.	Anxiety, depression, work stress (nursing profession/work score, time allocation/workload, working environment/equipment, patient nursing score, management/interpersonal), stress response (solve the problem, self-blame, ask for help, fantasize, retreat, rationalize), burnout, sleepiness	Nurses' work stress scale, Coping style questionnaire, Maslach burnout inventory, Pittsburgh sleep quality index, Self-rating anxiety scale, Self-rating depression scale
Goktas (2022)^43^ Turkey	Randomized controlled trial	Emergency department nurses (N = 60 (CG: 30, IG: 30))	CG: 28.70 (6.95), IG: 29.86 (7.56)	CG: 43.3% female, IG: 53.5% female	Motivational messages sent to nurses during Covid-19 pandemic.	No motivational messages.	Job satisfaction, compassion fatigue, Secondary trauma, occupational burnout	Compassion fatigue scale, Job satisfaction scale
Hata (2022)^44^ USA	Randomized controlled trial	Physicians, nurse practitioners, and certified nurse midwives (N = 23 (CG: 10, IG: 13))	NR	CG 91% female, IG 100% female	Monthly self-facilitated group meetings with structured discussion guide.	Monthly self-facilitated group meetings with no discussion guide or structure.	High depersonalization, high emotional exhaustion, overall high burnout, engagement, continuous burnout, empowerment at work, reaction to uncertainty, feel that department is committed to faculty wellbeing, feel sense of connection and community at work	Unknown instrument, physicians reaction to uncertainty scale, Maslach burnout inventory, Utrecht work engagement scale
Kose (2022)^47^ Turkey	Randomized controlled trial	ICU nurses (N = 87 (CG: 46, IG: 41))	Age CG: 26.9 (3.7), IG: 28.4 (7.6)	CG: 73.9% female, IG: 80.5% female	Nurses received 5–10 min breaks approved by the head nurse after message alerts. Motivational messages were sent to their phones daily at 09:00, 12:00, 17:00, and 19:00 for 21 days in the motivational group.	Nurses in the control group took breaks in accordance with their unit without messages.	Hopelessness, satisfaction with life, life orientation	Beck hopelessness scale, Life orientation test, Satisfaction with life scale
Ramanan (2020)^62^ USA	Before/after study	Neurology physicians (N = before: 21, after: 25)	NR	NR	The Resident Wellness Committee, co-chaired by a resident and staff neurologist, aimed to promote well-being through work-life integration, emotional and physical health, and social engagement.	No resident Wellness Committee.	Overall wellness	Self-devised
Ricou (2020)^60^ Switzerland	Randomized controlled trial	ICU nurses (N = 83 (CG: 42, IG: 41))	Age <40 CG: 72%, age <40 IG: 69%	CG: 80% female, IG: 78% female	Psychological support group sessions led by two psychologists for ICU teams during working hours	No psychological support sessions.	Burnout, hospital anxiety, hospital depression	Hospital anxiety and depression scale, Maslach burnout inventory
West (2014)^76^ USA	Randomized controlled trial	Physicians (N = 424 (CG: 37, IG: 37, non-study cohort: 350))	NR	CG: 35.1% female, IG: 32.4% female	The intervention included 19 biweekly physician discussion groups over 9 months, focusing on mindfulness, reflection, and shared learning. Participants received 1 h of paid time every other week. Sessions covered topics like self, patient, and balance, with check-ins, discussions, skill learning, and summaries.	Both trial arms received 1 h of protected time. In the control arm, participants could schedule and use this hour as they saw fit, without participating in discussion groups.	Engagement in work, overall burnout, stress, depression, overall quality of life, job satisfaction	Maslach burnout inventory, Perceived stress scale, Physician job satisfaction scale, Positive depression screen, VAS, Empowerment at work scale
Social resources & support | (additional) staff support								
Bakhru (2019)^36^ USA	Randomized observation study	ICU fellows and faculty (N = 33 (13 fellows, 20 faculty))	Faculty median: 38.5 (range 36.5–14), fellow median: 32 (range 30–33)	Faculty 30% female, fellows 61.5% female	Nighttime staffing model. In the intervention, there were in-hospital residents with an in-hospital nighttime intensivist.	During the standard staffing model, there were in-hospital residents, with a fellow and faculty member available at nighttime by phone.	Falling asleep, trouble overnight being awaked and unable to fall asleep, waking up, quality of sleep last night, current feeling in AM, KSS sleepiness, alertness, stress, happiness, sickness, physical exhaustion, mental exhaustion	VAS, Pittsburgh sleep quality index
Clubbs (2019)^39^ USA	Quasi-experimental and before/after study	NICU nurses (N = before: 25, after: 32)	NR	NR	A volunteer-based developmental care partner program ran from November 2014 to October 2015, ensuring infants received necessary sensory exposures for brain development. Volunteers, including students and faculty, committed to at least one weekly shift and 50 contact hours per year.	No volunteers/informal cuddlers.	Burnout (emotional exhaustion, personal accomplishment, depersonalization), infections infants (relative risk, absolute risk difference, preventive fractions, number needed to treat, exposed cases impact number, incident rates)	Maslach burnout inventory, electronic patient record data
Zhu (2020)^78^ USA	Before/after study	Acute care unit nurses and residents (N = before: 121 nurses; 6 residents, after: 155 nurses; 40 residents)	NR	NR	The nightly huddle involved: 1) a meeting between the night float resident and charge nurse to discuss clinical concerns and action plans; 2) bedside evaluations of reported patient issues; and 3) a review of non-urgent nursing requests using a standardized checklist.	No nightly huddle.	Quality of teamwork, communication, quality (has the nurse staff ever failed to notify you regarding important pt care info), understanding of patients plan, rating of timeliness in resident response	Self-devised
Edwards (2024)^83^ USA	Randomized controlled trial	Pediatric endocrinologists and developmental-behavioral pediatricians (N = 4)	NR	Gender: 75% female	Presence of a medical scribe.	No medical scribe.	Burnout (emotional exhaustion, personal accomplishment, depersonalization)	Maslach burnout inventory
Social resources & support | optimizing teams								
Ahn (2021)^34^ South Korea	Quasi-experimental study	Perioperative nurses (N = 60 (CG: 32, IG: 28))	28.25 (4.48)	96.7% female	The Teamwork Improvement Program included 4 sessions (60 min each) and web-based learning for 4–5 nurses per team. Activities involved teamwork games, discussions, and simulations. Modules covered team structure, communication, leadership, situation monitoring, mutual support, and a summary.	Control group receives no intervention.	Teamwork competencies (knowledge, attitudes, communication self-efficacy, skills and behavior), experience of surgical nursing errors	Learning benchmarks, Teamwork attitudes questionnaire, Teamwork perceptions questionnaire, Unknown survey
Amiri (2018)^33^ Iran	Randomized controlled trial	ICU nurses and supervisors (N = 61 (CG: 31, IG: 30))	33.46 (7.91)	86.9% female	Educational empowerment program. 2-day workshop (8 h). Hanging posters geared towards patient safety, patient safety culture, speaking out in situations of threat to patient safety and skills of the Team Strategies and Tools to Enhance Performance and Patient Safety, including communication, leadership, mutual support, situational monitoring skills.	Control group receives no intervention.	Teamwork within units, manager expectations and actions promoting patient safety, organizational learning and continuous improvement, management support for patient safety, overall perception of patient safety, feedback and communication on errors, communication openness, frequency of events reported, teamwork across hospital units, staffing, handoffs and transitions, Non-punitive response to errors, total scores of patient safety culture, safety score	Hospital survey on patient safety culture, One-item patient safety
Gausvik (2015)^41^ USA	Non-randomized controlled trial	Nurses, social workers, physical and occupational therapists, and patient care assistants (N = 62 (CG: 38, IG: 24))	NR	NR	Structured interdisciplinary bedside rounds on an acute care for the elderly unit.	The control group units utilized traditional physician-centric rounding.	Teamwork, understanding of plan, addresses fears/worries, team communication, family communication, efficiency, safety, job satisfaction	Self-devised
Personal development & recovery | role opportunities								
Keenan (2018)^46^ Canada	Before/after study	Neurosurgery nurses (N = before: 31, after: 41)	NR	NR	Two nurse practitioners were appointed to advanced practice roles with clinical duties, including health assessments, ordering tests and medications (excluding benzodiazepines and opioids), and discharging patients. They collaborated with neurosurgery residents, led family meetings, and managed discharge planning. The APNs focused 80% on clinical work and divided the remaining 20% between education, research, and leadership.	No advanced practice nurse positions.	Communication, learning opportunities	Self-devised
Shamsi (2016)^67^ Iran	Quasi-experimental and before/after study	Emergency department nurses (N = 35)	31.63 (7.7)	31.4% female	The “Stabilization care delivery model” starts when a patient is admitted to the emergency department and continues until stabilization. Patients are categorized into five groups, and nurses are assigned to five groups based on their specialties and interests.	No stabilization model.	Length of stay, job satisfaction	Mohr man-Cooke-Mohr man job satisfaction scale, length of stay with a chronometer
Personal development & recovery | relax opportunities								
Zion (2019)^79^ Israel	Before/after study	Nurses (N = 109)	39.0 (9.1)	100% female	Scheduled 30-min nap during an 8-hr night shift (23:00–7:00).	No powernap.	Subjective sleepiness	Karolinska sleepiness scale
Stevens (2020)^69^ USA	Non-randomized cross-over trial	Otolaryngology physicians (N = 19)	NR	47% female	Participants were allocated 2 h per week of protected nonclinical time. This time could be used for work-related administrative tasks or personal health and well-being activities.	No 2 h per week of protected time.	Burnout (emotional exhaustion, depersonalization, personal accomplishment), wellbeing, quality of life, job satisfaction, job stress, burnout, control over workload, sufficient time for documentation, time spent at home on EMR	Mini-Z survey, Maslach burnout inventory, One-item quality of life, Well-being index
Personal development & recovery | other team/setting opportunities								
Zhong (2022)^77^ China	Non-randomized controlled trial	Operating room nurses (N = 5371 (CG: 2474, IG: 2891))	NR	NR	Fixed nurse teams are defined as operating room nurse teams within a surgical subdiscipline that works together for an extended duration, typically at least one year.	Non fixed nursing teams.	Quality (surgical patient assessment rate, surgery location mark assessment rate, allergy history assessment rate, rate of assessing antibiotics use 60 min before incision, sterilization indicator results assessment rate, surgical equipment and surgical materials availability rate, surgery name confirmation rate, surgical tools inventory rate, surgical specimen checking rate, postoperative surgical equipment inspection rate, patient acute pressure ulcer rate during surgery, rate of leaving surgical foreign objects behind, rate of perioperative drug use, transfusion reaction rate during the surgical period, unplanned extubation rate, incidence of needle punctures among medical personnel, incidence of surgical patients falling or falling out of bed, incidence of electrical burns, incidence of surgical site infections), job satisfaction	Rating scale, Unknown instrument
Byrne (2020)^38^ Australia	Non-randomized controlled trial	Nurses and midwives (N = 54 (CG: 29, IG: 24))	NR	NR	An exchange program paired rural/remote nurses or midwives with metropolitan/regional counterparts for a professional job swap, with options for three or six-month exchanges.	No exchange program.	Job Satisfaction, turnover intention, occupation attrition, burnout, global health, leadership, network adequacy, community fit, community sacrifice, organizational fit, organizational sacrifice	Rating scale, General health questionnaire, Burnout measure—short version, Job embeddedness measure, Turnover intention scale
Multi-categorical								
Petrie (2022)^59^ Australia	Before/after study	Physicians (N = before: 279, after: 344)	Before: 21–30: 46.2%, 31–40: 29.5%, 41–50: 11.4%, 51–60: 8.9%, >61: 4% vs. 5%, after: 21–30: 44.1%, 31–40: 26.7%, 41–50: 14%, 51–60: 10.2%, >61: 5%	Before: 47.2% female, after: 46.6% female	The multi-model intervention included nine strategies: 1) Hiring an additional junior doctor to reduce overtime; 2) Establishing the Doctors Wellness Committee with 5 meetings annually; 3) Streamlining overtime claims; 4) Conducting the 2017 Doctors Wellness Survey; 5) Organizing a wellness forum; 6) Presenting mental health sessions; 7) Offering mental health training for staff; 8) Providing mandatory mental health training for doctors-in-training; 9) Implementing mentoring and peer support programs for interns.	Control period without multi-modal intervention.	Overall job satisfaction, work-related stressors, work-life balance, workplace support, workload, workplace bullying and harassment, psychological distress, suicidal ideation	Medicine in Australia: Balancing employment and life, self-devised, Kessler psychological distress scale

NR: not reported; IG: intervention group; CG: control group; ICU: intensive care unit; SD: standard deviation; PDCA: Plan Do Check Act; EU: European Union; CES-D: Center for epidemiologic studies depression scale; ESI: Emergency severity index; KAP: Knowledge, attitude, and practice model; NICU: Neonatal intensive care unit; NASA: National aeronautics and space administration; SF-36: Short form health survey; VAS: Visual analog scale; KSS: Karolinska sleepiness scale; Db: Decibel.

Altogether 419 outcomes were extracted and cleaned by AB and KvdB following the JD-R categories: (a) Job demands (workload, misconduct); (b) Job resources (communication, support, team climate); (c) Leadership; (d) Personal resources (concentration, coping, efficiency, emotions, psychological characteristics); (e) Well-being (anxiety, burnout, depression, general health, job satisfaction, lack of energy, physical discomfort, quality of life, sleep, stress); (f) retention outcomes (employability/engagement/turnover intentions); (g) Quality/safety outcomes (patient quality, patient safety, unwanted events).

One study was judged to be at overall low risk of bias, as all ratings were assessed as ‘low risk’.^44^ Remaining studies were all considered to have some risk of bias. Two studies received one ‘unclear’ or ‘high risk’ rating.^65^^,^^66^ Four studies received two ‘unclear’ or ‘high risk’ ratings.^34^^,^^43^^,^^47^^,^^70^ Other studies received more than two ‘unclear’ or ‘high risk’ ratings (detailed in Additional File 3). The GRADE methodology shows that evidence is of very low certainty. For two outcomes, burnout and job satisfaction, the certainty of evidence for *social resources & support* interventions was rated as low rather than very low. For further explanation and insight into the GRADE tables, see Additional File 4.

Results are presented per outcome category. Table 2 shows effect directions per outcome and intervention type for critical outcomes selected by the WHO as most relevant. Effect directions, statistical significance of effects (significant, non-significant, unclear significance), and unclear effects are determined and visualized. Forest plots are provided for eligible outcomes (Additional File 5: Supplementary plots (plot A Workload; plot B Support; plot C Team Climate; plot D Burnout; plot E Depression; plot F Job satisfaction; plot G Employability; plot H Patient safety)). A complete overview of effect directions is shown in Additional File 6. The effect direction table encompassing interventions evaluated on nurses are presented in Additional File 7 and interventions evaluated on doctors are presented in Additional File 8. A description of results on outcomes deemed important but not critical are given in Additional File 9.

**Table 2:** Effect direction table.

### Job demands

This domain contains workload (critical) and misconduct (important) outcomes. Results for the misconduct outcomes can be found in Additional Files 6 and 9.

#### Workload

Eleven studies measured workload outcomes,^51^^,^^56^^,^^59^^,^^66^^,^^71^^,^^73^^,^^80^, ^81^, ^82^^,^^84^^,^^85^ using ten different measurements (e.g., NASA Task Load Index, 5-item job demands scale, numeric rating scales, SWAT questionnaire) of which two outcome measures were not reported. One study on ‘workhours’ significantly favored the intervention evaluating 12-h shifts.^84^ One study on a *multi-categorical* intervention showed a significant effect versus no intervention.^59^ Eight studies from seven intervention categories were meta-analyzed (plot A).^56^^,^^59^^,^^66^^,^^73^^,^^80^, ^81^, ^82^^,^^84^ Given the variation in effect direction but overlapping confidence intervals within intervention categories, the effects on workload show moderate heterogeneity with SMDs from −1.09 to 0.39.

### Well-being

All outcomes within this outcome domain were deemed critical and are presented below.

#### Anxiety

Three studies measured anxiety outcomes,^51^^,^^60^^,^^71^ including three questionnaires (e.g., Hospital anxiety and depression scale, State-trait anxiety inventory-state). Two significantly favored the intervention: one ‘environment’ intervention (music versus no music during operations),^71^ and an ‘emotional support’ intervention (rational emotional intervention, integrated with hierarchical management, involved weekly 1-h sessions versus no intervention).^51^

#### Burnout

Nineteen studies measured burnout outcomes,^35^^,^^38^^,^^39^^,^^43^^,^^44^^,^^49^, ^50^, ^51^, ^52^^,^^58^^,^^60^^,^^61^^,^^64^^,^^66^^,^^69^^,^^76^^,^^80^^,^^83^^,^^84^ using seven questionnaires with the Maslach burnout inventory being most commonly. Eights significantly favored the intervention,^35^^,^^39^^,^^43^^,^^44^^,^^49^, ^50^, ^51^^,^^84^ including three ‘workhours’ interventions (12-h shifts versus 7/8-h shifts and duty-hour regulations versus no regulations),^35^^,^^50^^,^^84^ one ‘equipment support & patient handling’ intervention (video versus nurse education),^51^ three ‘emotional support’ interventions (rational emotional intervention, integrated with hierarchical management, involved weekly 1-h sessions, motivational messages versus no intervention and self-facilitated group meetings with and without structured discussion guide),^43^^,^^44^^,^^51^ and one ‘staff support’ intervention (volunteers/informal cuddlers versus no intervention).^39^ Two studies significantly favored the control groups^52^^,^^61^ (4-week rotations versus 2-week rotations,^52^ EU Working Time Directive versus no intervention^61^). Six studies were appropriate for meta-analysis (plot D),^35^^,^^49^^,^^58^^,^^61^^,^^66^^,^^80^ showing heterogeneous effects given various effect directions and non-overlapping confidence intervals within intervention categories with SMDs from −0.59 to 1.52. Few studies illustrate improved burnout, with some interventions illustrating contradicting effects.

#### Depression

Nine studies measured depression outcomes,^37^^,^^50^^,^^51^^,^^54^^,^^60^^,^^66^^,^^68^^,^^73^^,^^76^ using six questionnaires (e.g., Hospital and depression scale, Beck depression inventory). One significantly favored an ‘emotional support’ intervention (rational emotional intervention, integrated with hierarchical management, involved weekly 1-h sessions versus no intervention).^51^ Five studies were appropriate for meta-analysis (plot E),^37^^,^^54^^,^^66^^,^^68^^,^^73^ showing moderate heterogeneity with variation in effect direction but overlapping confidence intervals within intervention categories with SMDs from −0.44 to 0.64. None of the interventions significantly improved depression.

#### General health

Eight studies measured general health outcomes,^37^^,^^38^^,^^48^^,^^50^^,^^62^^,^^63^^,^^69^^,^^74^ using eight questionnaires, e.g., on wellness/feeling good (e.g., Wellbeing index, Short form health survey). One significantly favored an ‘environment’ intervention (Luminette light glasses versus no intervention).^37^

#### Job satisfaction

Seventeen studies measured job satisfaction,^38^^,^^40^, ^41^, ^42^, ^43^^,^^45^^,^^53^^,^^55^^,^^59^^,^^57^^,^^66^^,^^67^^,^^69^^,^^75^, ^76^, ^77^^,^^85^ using fifteen questionnaires (e.g., Physician job satisfaction scale, Global measure of work satisfaction). Nine significantly favored the interventions,^41^^,^^43^^,^^45^^,^^53^^,^^57^^,^^59^^,^^67^^,^^75^^,^^77^ including one ‘workhours’ (8.5-h shifts versus 12-h shifts),^75^ two ‘continuous improvement’ (PDCA-cycle management),^53^^,^^57^ one ‘workflow’ (hospital-based primary-care center versus traditional emergency department),^45^ one ‘emotional support’ (motivational messages versus no intervention),^43^ one ‘optimizing teams’ (interdisciplinary versus physician bedside rounds),^41^ one ‘role opportunities’ (stabilized care delivery versus no intervention),^67^ one ‘other team/setting opportunities’ (fixed-nursing teams versus non-fixed nursing teams),^77^ and one *multi-categorical* intervention (nine strategies versus no intervention).^59^ Five studies were appropriate for meta-analysis (plot F),^40^^,^^42^^,^^45^^,^^57^^,^^66^ showing heterogeneous effects given various effect directions and non-overlapping confidence intervals within intervention categories with SMDs from −0.46 to 3.56. One ‘continuous improvement’ intervention significantly favored the control,^66^ while another ‘continuous improvement’ significantly favored the intervention.^57^

#### Lack of energy

Ten studies measured lack of energy outcomes,^36^^,^^37^^,^^50^^,^^51^^,^^56^^,^^58^^,^^64^^,^^68^^,^^79^^,^^81^ using ten questionnaires, e.g., on sleep/fatigue/exhaustion (e.g., Fatigue assessment scale, Epworth sleepiness scale). Five significantly favored the intervention,^36^^,^^37^^,^^51^^,^^58^^,^^79^ including one ‘workhours’ intervention (16-h shifts versus 24-h shifts),^58^ one ‘environment’ intervention (Luminette light glasses versus no intervention),^37^ one ‘emotional support’ intervention (rational emotional intervention, integrated with hierarchical management, involved weekly 1-h sessions versus no intervention),^51^ one ‘staff support’ intervention (in-hospital nighttime intensivist versus intensivists available by phone),^36^ and one ‘relax opportunities’ intervention (nightly powernap versus no powernap).^79^

#### Physical discomfort

Six studies measured physical discomfort outcomes,^35^^,^^37^^,^^56^^,^^63^^,^^81^^,^^86^ using seven questionnaires, e.g., on back/shoulder/wrist/knee/back pain (e.g., Nordic questionnaire). One significantly favored an ‘environment’ intervention (Luminette light glasses versus no intervention).^37^

#### Quality of life

Four studies measured quality of life,^47^^,^^50^^,^^68^^,^^69^^,^^76^ using four questionnaires (e.g., Satisfaction with life scale, single-item assessment). One significantly favored an ‘emotional support’ intervention (motivational messages versus no intervention).^47^ Another study effect favored the control group (standard light settings) over high-intensity dynamic light.^68^

#### Sleep

Six studies measured sleep outcomes,^36^^,^^50^^,^^68^^,^^75^^,^^81^^,^^85^ using six questionnaires (e.g., Pittsburgh sleep quality index). Two significantly favored interventions: one ‘workhours’ (8.5-h shifts versus 12-h shifts),^75^ and one ‘staff support’ (in-hospital nighttime intensivist versus phone ability).^36^

#### Stress

Ten studies measured stress outcomes,^36^^,^^42^^,^^43^^,^^49^^,^^52^^,^^59^^,^^69^^,^^76^^,^^80^^,^^82^ using ten questionnaires (e.g., perceived stress scale, Kessler psychological distress scale). Two significantly favored the interventions: one ‘equipment support & patient handling’ (video versus nurse education),^49^ and one ‘emotional support’ (motivational messages versus no intervention).^43^ One study significantly favored the control (2-week rotations) over the intervention (4-week rotations).^52^

### Retention outcomes

All outcomes within this retention outcome domain were deemed critical and are presented below.

#### Employability

Thirteen studies measured employability,^35^, ^36^, ^37^^,^^44^^,^^55^^,^^61^^,^^63^^,^^65^^,^^72^^,^^74^^,^^75^^,^^80^^,^^86^ using thirteen measurements (e.g., hospital data, self-rated sickness/productivity/performance). The Luminette light glasses intervention significantly improved employability.^37^ Two study effects significantly favored the control group (EU Working Time Directive versus no intervention, 12-h shifts versus 8-h shifts^61^^,^^65^). Six studies were appropriate for meta-analysis (plot G),^36^^,^^37^^,^^44^^,^^61^^,^^74^^,^^80^ showing moderately heterogeneous effects given variation in effect direction but overlapping confidence intervals within intervention categories with SMDs from −0.26 to 0.66.

#### Engagement

Four studies measured engagement outcomes,^38^^,^^44^^,^^76^^,^^80^ using four questionnaires (e.g., Job embeddedness measure, Utrecht work engagement scale). Two significantly favored ‘emotional support’ interventions (group meetings).^44^^,^^76^

#### Turnover intention

Three studies measured turnover intention outcomes,^38^^,^^66^^,^^75^ using four questionnaires (e.g., single-item assessment, 3-item turnover intention scale). None favored the intervention. One significantly favored the control group (no intervention) over solution meetings to discuss stressors.^66^

### Quality & safety of care

All outcomes within this outcome domain were deemed critical and are presented below.

#### Patient quality

Five studies measured quality of care outcomes,^50^^,^^67^^,^^70^^,^^77^^,^^78^ using five measurements for quality indicators as length of stay and drug delivery time (four self-developed). Two significantly favored the intervention,^67^^,^^78^ specifically ‘staff support’ (nightly huddle to discuss patients versus no intervention), and ‘role opportunities' (stabilization care model versus no intervention).

#### Patient safety

Six studies measured patient safety outcomes,^33^^,^^41^^,^^66^^,^^85^^,^^86^ using eight measurements for perceptions on patient safety, experienced patient safety culture and handling (e.g., Hospital survey on patient safety culture). Three significantly favored the intervention^33^^,^^41^^,^^86^: one ‘equipment support & patient handling’ (safe patient handling and mobilization program versus no intervention),^86^ and two ‘optimizing teams’ interventions (empowerment program versus no intervention and interdisciplinary versus physician bedside rounds).^33^^,^^41^ Four studies were appropriate for meta-analysis (plot H),^33^^,^^41^^,^^66^^,^^86^ showing heterogeneous effects given the various effect directions and non-overlapping confidence intervals within intervention categories with SMDs from −0.23 to 1.91.

#### Unwanted event

Eleven studies measured unwanted event outcomes,^33^^,^^34^^,^^39^^,^^40^^,^^42^^,^^58^^,^^63^^,^^66^^,^^70^^,^^75^^,^^77^ using ten methods (e.g., hospital data, self-rated accidents/errors/infections/patient falls/readmissions). None significantly favored the interventions.

### Job resources, leadership, and personal resources

The outcomes of the Job resources, Leadership, and Personal resources domains were all deemed important but not critical. Their results can be found in Additional Files 6 and 9. The outcomes within the Job resources domain: communication, job control, support, and team climate. Personal resources outcomes were concentration, coping, efficiency, emotions, and psychological characteristics.

## Discussion

Our research highlights the focus on HCP well-being in recent years. However, we noted a wide variety of described interventions targeting numerous outcomes using diverse instruments. When synthesizing these interventions, some show improvements in well-being, while others may be detrimental.^66^ Certain interventions might be effective in one aspect of well-being but have negative effects on another, complicating intervention selection. Therefore, careful consideration of multiple factors is crucial for making effective decisions.

Interventions focusing on *social resource & support* showed the most consistent significant improvements over a variety of outcome categories based on the effect direction table (e.g., rational emotional intervention). This result may be related to the fact that these interventions align with HCP' needs (recognizing, personal attention and support), making them practical and adaptable to local contexts, which are features of ideal workplace interventions within healthcare organizations.^88^ Understanding target groups, their fears, preferences and fulfilling their needs are key issues in intervention development.^89^^,^^90^

*Personal development & recovery* interventions showed mixed results: ‘role opportunities’ were effective, while ‘relax opportunities’ and ‘other team/setting opportunities’ were less so. Apart from one study, sample sizes, study designs, settings, and HCPs were similar. Inconsistencies in effects may stem from manual intervention categorization. Interventions like the stabilization care model,^67^ advanced practice nurses,^46^ and nightly 30-min naps^79^ have shown favorable effects on job satisfaction, quality of care, and reducing unwanted events. In contrast, interventions such as protected nonclinical time,^69^ fixed nursing teams,^77^ and exchange programs^38^ showed no significant effects or lacked sufficient information to judge their effectiveness. Further research is needed to explore underlying mechanisms to understand and explain when and why interventions work or not. Inconsistencies may arise from interventions not effectively addressing underlying problems.^89^

Within the intervention type *Management & building*, the category ‘workhours’ show also mixed results, however this explanation differs. Within this example mixed results appear possibly due to variety in the comparator interventions. Four studies compare 12-h shifts.^65^^,^^75^^,^^81^^,^^84^ Three studies described 12-h shifts as the intervention,^65^^,^^81^^,^^84^ while another study describes 12-h shifts as the control.^75^ Moreover, the comparator also differs, as 12-h shifts are compared with, for example, 7-h^84^ and 8-h^35^ shifts, demonstrating that the local context is essential to consider. Overall, considering the studies comparing shift lengths (7-, 8-, 12-, 16-, and 24-h shifts), shorter shifts appear preferable in four^58^^,^^65^^,^^75^^,^^81^ out of six studies.^35^^,^^58^^,^^65^^,^^75^^,^^81^^,^^84^ Starting from a different baseline impact results significantly. Inconsistency might also be due to group sizes and study quality. Interventions, e.g., in the ‘emotional support’ category, high bias or small samples might led to less significant results.

We identified several studies suggesting that certain interventions may have harmful effects on some aspects of well-being. For example, in studies on moderated solution meetings^66^ and high-intensity dynamic light,^68^ outcomes such as job satisfaction, turnover intention, and quality of life were significantly better in the control groups. This finding is important, as it suggests that not receiving the intervention may sometimes be more beneficial. Potential mechanisms of harm were rarely explored, but may include a lack of alignment with staff needs, or contextual factors such as organizational restructuring or construction plans mentioned.^66^ Moreover, well-being is a multidimensional concept, and we applied the JD-R model, which considers various outcomes (influencing factors as workload, intermediate outcomes as job satisfaction, and final consequences as turnover intention). This may explain diverging effect directions. For example, an intervention that increases efficiency (positive for performance and an example of a job resource) may also raise workload (negative for well-being, related to job demands). Similarly, moderated solution meetings^66^ may enhance job control but reduce job satisfaction, possibly because professionals find these non-patient-related responsibilities less enjoyable.

We found sixteen interventions evaluated solely among doctors and 24 among nurses. Unlike nurses, no interventions for doctors targeted optimizing teams, or role/setting opportunities. Unlike doctors, no interventions for nurses targeted on multi-categorical interventions. Interventions on job demands are more often studied in doctors, those on job resources in nurses.

Examplesof effective interventions include Bragard's study^37^ on Luminette light glasses, which improved general health, energy, physical discomfort, and employability. Liu's hierarchical management sessions^51^ enhanced coping, anxiety, burnout, depression, and energy. Goktas' motivational messages^43^ reduced burnout, stress, and increased job satisfaction. Hata's monthly group meetings^44^ improved team climate, burnout, and engagement. Bakhru's nighttime intensivists^36^ improved concentration, energy, and sleep. Gausvik's interdisciplinary bedrounds^41^ enhanced communication, team climate, efficiency, job satisfaction, and patient safety. Petrie's multi-model intervention^59^ improved misconduct, workload, support, and job satisfaction. These positive effects may be explained by contextual factors, such as a setting with high problem pressure and therefore high potential for improvement. For example, Liu^51^ and Goktas^43^ mentioned high workload in the ED, a young nursing workforce, and the COVID-19 period, during which job satisfaction was generally lower and anxiety higher.

Besides adherence, implementation success and involvement of HCP themselves,^91^ when healthcare leaders and/or organizations aim to improve HCP' well-being by selecting the most appropriate evidence-based interventions, it is important to consider how data on their well-being have been captured. For example, when the burnout rate was obtained through the human resource department, then the burnout rate is valid. However, if the rate was collected from a well-being survey, the response rate might introduce bias, as only a selective group may have participated, possibly skewing results toward those with burnout issues. Organization-directed interventions could benefit survey respondents but might not help, or could harm, non-respondents who may not have burnout issues.

Understanding outcome's cause is crucial, as it may be work-related or non-work related. In some populations, non-work-related burnout may be more prevalent.^92^ Organization-directed interventions, e.g., targeting work-related burnout (e.g., adjusting work hours) might be ineffective if burnout is non-work-related. However, interventions for non-work-related issues (e.g., free counseling) could help. Population characteristics, such as gender distribution and job roles (nurses vs. doctors), also influence intervention effectiveness. This variability contributes to observed heterogeneity in interventions and outcomes. The direction table is split out for nurses and doctors in Additional Files 7 and 8.

Providing straightforward recommendations for leaders selecting right interventions is challenging due to the high heterogeneity in interventions and outcomes. Selection of interventions should be preceded with an in-detail analysis of the root causes of the problem and an assessment of HCPs' needs for which various instruments exist^93^ That assessment is a common issue and that this step is indeed missing or receives insufficient attention is also evident from a recent published review on organizational interventions,^91^ which observed that studies did not address issues directly but focused on secondary prevention. If the intended issue is clear, Table 2 can help determine possibly fitting interventions. According to the JD-R model, improving HCPs' well-being and ultimately their retention and performance requires enhancing (job) resources while reducing job demands.^28^ In a context where job demands are high, the availability of sufficient resources can mitigate their negative effects.^28^ Conversely, when job demands are high and resources are limited, the risk of burnout and turnover is greatest. This interaction underscores that interventions to enhance HCPs' well-being and retention should simultaneously reduce job demands and strengthen resources.^28^ All interventions in this study are classified as work-related through the lens of the JD-R model, focusing on tweaking the work environment rather than personal resources. Intervention like *management & building, social resources & support, and (facilitating) personal development & recovery* can enhance job resources, buffering the negative effects of job demands and improving well-being. Interventions that reduce workload decrease job demands, alleviating stress and burnout, and enhancing well-being. The combination of both organizational-directed interventions and individual-directed interventions is a known successful concept.^91^ Performing multi-level interventions could promote HCPs' well-being.^91^ Within this process, organizations must carefully consider potential positive and negative effects. Interventions, e.g., 12 vs. 8-hour work shifts showed significant positive effects on burnout (Table 2) but may decrease employability. Thus, “if it does not help, it does not hurt” does not apply. Table 2 helps weigh potential effects but also highlights gaps in information on essential outcomes (empty cells), complicating decision-making. Furthermore, it is evident that selecting the right intervention needs to be followed by implementation, adaptation, and (long-term) evaluation to reach maximal impact and quality improvement.^94^

To make better informed decisions with more certainty and less data heterogeneity, consensus on aspects of HCP' well-being and their measurement is needed. Some interventions reported only positive outcomes, yet many well-being outcomes were unassessed (as shown by empty cells in Table 2). For effective decision-making, information on potential negative impacts is crucial. Developing a core outcome set reflecting positive and negative effects of interventions on well-being is essential. To interpret broader system-level impacts, organizational outcomes and contextual moderators should be considered for future research.

This review highlights the importance and effectiveness of organization-directed interventions. It emphasizes organization-directed over individual-directed interventions and includes a wide range of outcomes. Meta-analyses were conducted where feasible, with effect-direction Tables for other results to maximize data utilization. Moreover, the study followed the registered protocol, using pre-established criteria ensuring systematic conduct. Categories were determined inductively instead of using the predetermined non-supportive types.

This study also holds some limitations. First, data screening and extraction by different researchers may introduce interpretation variations, despite calibration attempts. However, the procedure we followed with the high number articles identified was unavoidable. Furthermore, determining the eligibility of interventions was at times challenging. For instance, we included interventions delivered to teams^60^ but excluded those primarily targeting individuals.^95^^,^^96^ This distinction was complicated by the fact that some team-level interventions, such as motivational messages or protected nonclinical time, may still affect individual behavior. Nevertheless, we believe these decisions did not significantly affect study selection or interpretation, due to the iterative discussions and consistent application of predefined criteria^12^ by the research team. Additionally, interventions with an initial strong focus on efficiency rather dan well-being were excluded.^97^^,^^98^ For transparency, we included an overview of screened full-text articles excluded with corresponding reasons in Additional File 10. Second, the diverse reporting of outcomes, often measured using different or non-validated instruments, posed challenges in categorization and interpretation. Some constructs, such as stress, anxiety, and burnout, may overlap conceptually, and their inclusion in separate categories may lead to redundancy. We addressed this by relying on the primary construct identified by the study authors or measurement tools, but ambiguity remains. Moreover, not all instruments were validated, which limits comparability and reliability. These issues highlight the need for caution in interpreting the synthesized outcomes and call for greater standardization in future research. Furthermore, the primary aim was to synthesize the effects of organizational-level interventions on healthcare professionals' well-being, guided by the JD-R model. As such, we focused our outcome extraction on indicators of individual well-being at work, rather than organizational performance metrics such as system efficiency. This may constrain the interpretation of broader system-level impacts. Third, the meta-analyses included heterogeneous data and most of the included studies use observational, before-after, or quasi-experimental designs, complicating analyses (despite the data being split at different levels and sub-groups), conclusions, and generalizability. This limits the ability to draw strong causal inferences. However, meta-analysis was only performed on an exploratory basis with at least five studies, categorized by intervention type.^32^ Moreover doubtful cases were excluded from the meta-analysis. Although the inclusion of a broad range of interventions and outcomes introduces heterogeneity, it reflects the current diversity in the field and supports our aim to map the full range of organization-directed approaches being explored.

Lastly, databases like PsychINFO were excluded because they primarily focus on psychological and behavioral sciences. This decision may have resulted in some studies being missed. Future research should identify relevant situations and objectives for interventions, evaluate multi-type intervention bundles with larger sample sizes, and tailor them to local contexts. Sharing and understanding intervention effects is crucial to ensure they benefit or at least do not harm HCPs' well-being.

This review highlights that organization-directed interventions can contribute to improving healthcare professionals' well-being, work environment, retention, and quality of care. A one-size-fits-all approach is not feasible. Interventions focused on social resources and support consistently yielded positive effects across different outcome domains. This pattern should be interpreted with caution due to the overall low quality of the evidence, including the fact that only one study was rated as low risk of bias. Furthermore, the overall findings are mixed. Some interventions demonstrated beneficial effects for certain outcomes while showing neutral or even detrimental effects for others. These variations underscore the importance of further research with more rigorous study designs, larger sample sizes, a consistent core outcome set, conducting a thorough needs assessment in advance, understanding potential contributors to reduced well-being, and considering local contextual factors before selecting or implementing interventions.

## Contributors

Conceptualization: AB, KvdB, LH, AF, MvdL. Management and coordination: AB. Data collection: AB, KvdB. Analysis: AB, KvdB, LH. Writing original draft: AB. Writing (review & editing): KvdB. Supervision: LH, MO, AF, MvdL. Writing (reviewing): KvdB, LH, MO, AF, MvdL. Funding acquisition: LH, AF, MvdL. All authors read and approved the final version of this manuscript. AB and KvdB had direct access to and verified the underlying data. All authors agreed being accountable for all aspects of the work ensuring questions related to accuracy or integrity of any part of the work are appropriately investigated and resolved.

## Data sharing statement

All data relevant to the study are included in the article or uploaded as supplementary information.

## Declaration of interests

For this work AB, KvdB, LH, AF, and MvdL were supported by funders NFU and ZIN. MO has nothing to declare.
