# Supplementary material for: Effectiveness of organization-directed interventions on healthcare professionals' well-being: a systematic review
Source: eClinicalMedicine. 2025 Sep 13;88:103496. doi: 10.1016/j.eclinm.2025.103496 (PMC12572811; doi:10.1016/j.eclinm.2025.103496)
Supplement: Additional Files 1–10 [file mmc1.docx]

**Additional files**

**Content table**

| 1. Search strategy | Page 2 |
| --- | --- |
| 1. Themes and categories specified | Page 5 |
| 1. Risk of bias assessment | Page 6 |
| 1. GRADE tables | Page 8 |
| 1. Supplementary plots (plot A Workload; plot B Support; plot C Team Climate; plot D Burnout; plot E Depression; plot F Job satisfaction; plot G Employability; plot H Patient safety) | Page 8 |
| 1. Direction table complete | Page 12 |
| 1. Direction table nurses | Page 14 |
| 1. Direction table doctors | Page 16 |
| 1. Description of results on outcomes Job resources, Leadership and Personal resources | Page 17 |
| 1. An overview of screened full-text articles excluded with corresponding reasons | Page 20 |

**Additional file 1: search strategy**

| **#** | **Searches MEDLINE** | **Results** |
| --- | --- | --- |
|  |  |  |
| 1 | ("Healthcare professionals" or "Healthcare providers" or Practitioners or Doctor or nurse* or nursing or physician? or resident? or "healthcare worker" or "health staff").ti. or exp *"physicians"/ or exp *"Medical staff"/ or *"Residents"/ or exp *"Nurses"/ or exp *"Nursing Staff"/ | 604063 |
| 2 | exp Hospitals/ | 310449 |
| 3 | (inpatient* or hospital* or department* or ward or ICU or ((healthcare or health-care) adj cent*)).ti,ab,kf. | 1906733 |
| 4 | 2 or 3 | 2001371 |
| 5 | Personnel Turnover/ | 5826 |
| 6 | (((job or work) adj3 (satisfaction or culture or environment)) or work-load or workload or (sick adj2 leave) or absenteeism or presenteeism or ((turnover or leave) adj3 intention) or ((Employee or Personnel) adj3 (Turnover* or Retention*))).ti,ab,kf. | 74773 |
| 7 | exp Job Satisfaction/ | 28062 |
| 8 | exp Organizational Culture/ | 18866 |
| 9 | exp Workload/ | 23641 |
| 10 | exp Absenteeism/ | 9713 |
| 11 | exp Presenteeism/ | 561 |
| 12 | exp Burnout, Psychological/ or exp Personal Satisfaction/ or (((work or job) adj3 satisfaction) or workload or well-being or fulfilment or burnout or thriving).ti,ab,kf. | 187842 |
| 13 | Work-Life Balance/ | 1010 |
| 14 | exp Sick Leave/ | 6644 |
| 15 | 5 or 6 or 7 or 8 or 9 or 10 or 11 or 12 or 13 or 14 | 269119 |
| 16 | 1 and 4 and 15 | 14934 |
| 17 | exp Controlled Before-After Studies/ | 707 |
| 18 | exp Interrupted Time Series Analysis/ | 1730 |
| 19 | exp Randomized Controlled Trial/ | 583524 |
| 20 | exp Cluster Analysis/ | 71819 |
| 21 | exp Comparative Study/ | 1911827 |
| 22 | ((control* adj2 before) or CBA or (interrupted adj2 time-ser*) or (cluster adj2 (random* or RCT))).ti,ab,kf. or comparative.ti. | 232563 |
| 23 | 17 or 18 or 19 or 20 or 21 or 22 | 2526482 |
| 24 | 16 and 23 | 1138 |
| 25 | limit 24 to yr="2012 -Current" | 500 |
|  | Total | 733 |

| **#** | **Searches Embase** | **Results** |
| --- | --- | --- |
| #14 | #13 AND (2012:py OR 2013:py OR 2014:py OR 2015:py OR 2016:py OR 2017:py OR 2018:py OR 2019:py OR 2020:py OR 2021:py OR 2022:py) | 1382 |
| #13 | #12 AND [embase]/lim NOT 'conference abstract'/it | 1866 |
| #12 | #8 AND #11 | 3458 |
| #11 | #9 OR #10 | 10643505 |
| #10 | 'controlled study'/exp OR 'randomized controlled trial'/exp OR 'cluster analysis'/exp OR 'comparative study'/exp | 10532428 |
| #9 | ((control* NEAR/2 before):ti,ab,kw) OR cba:ti,ab,kw OR ((interrupted NEAR/2 'time ser*'):ti,ab,kw) OR ((cluster NEAR/2 (random* OR rct)):ti,ab,kw) OR comparative:ti | 287378 |
| #8 | #3 AND #4 AND #7 | 16256 |
| #7 | #5 OR #6 | 315714 |
| #6 | 'job satisfaction'/exp OR 'organizational culture'/exp OR 'workload'/exp OR 'absenteeism'/exp OR 'presenteeism'/exp OR 'burnout'/exp OR 'life satisfaction'/exp OR 'work-life balance'/exp OR 'medical leave'/exp | 145164 |
| #5 | (((job OR work) NEAR/3 (satisfaction OR culture OR environment)):ti,ab,kw) OR 'work load':ti,ab,kw OR workload:ti,ab,kw OR ((sick NEAR/2 leave):ti,ab,kw) OR absenteeism:ti,ab,kw OR presenteeism:ti,ab,kw OR (((turnover OR leave) NEAR/3 intention):ti,ab,kw) OR (((employee OR personnel) NEAR/3 (turnover* OR retention*)):ti,ab,kw) OR (((work OR job) NEAR/3 satisfaction):ti,ab,kw) OR 'well being':ti,ab,kw OR fulfilment:ti,ab,kw OR burnout:ti,ab,kw OR thriving:ti,ab,kw | 246825 |
| #4 | 'hospital'/exp OR inpatient*:ti,ab,kw OR hospital*:ti,ab,kw OR department*:ti,ab,kw OR ward:ti,ab,kw OR icu:ti,ab,kw OR ((healthcare NEAR/1 cent*):ti,ab,kw) OR (('health care' NEAR/1 cent*):ti,ab,kw) | 3460508 |
| #3 | #1 OR #2 | 650695 |
| #2 | 'health care personnel'/mj OR 'medical personnel'/mj OR 'nurse'/mj OR 'nursing staff'/mj | 137726 |
| #1 | 'healthcare professional*':ti OR 'healthcare provider*':ti OR practitioner*:ti OR doctor*:ti OR nurse*:ti OR nursing:ti OR physician*:ti OR resident*:ti OR 'healthcare work*':ti OR 'health staff':ti | 583435 |
|  | Total | 1382 |

| **#** | **Searches Cinahl** | **Results** |
| --- | --- | --- |
| S25 | S9 AND S16 AND S22 AND S23 (Limiters - Exclude MEDLINE records) | 1,329 |
| S24 | S9 AND S16 AND S22 AND S23 | 2,29 |
| S23 | S17 OR S18 OR S19 OR S20 OR S21 | 464,47 |
| S22 | S1 OR S2 OR S3 OR S4 | 421,434 |
| S21 | TI(satisfaction or well-being or fulfilment or burnout or ((psychological or mental) N1 health) or thriving or environment or ethic*) | 145,799 |
| S20 | AB(satisfaction or well-being or fulfilment or burnout or ((psychological or mental) N1 health) or thriving or environment or ethic*) | 365,66 |
| S19 | (MH "Mental Health") | 44,106 |
| S18 | (MH "Job Satisfaction") OR (MH "Personal Satisfaction") | 35,259 |
| S17 | (MH "Burnout, Professional") | 12,506 |
| S16 | S11 OR S12 OR S13 OR S14 OR S15 | 502,496 |
| S15 | TI(valid* or (cronbach* N3 (alpha or alphas)) or interrater or inter-rater or intrarater or intra-rater or intertester or inter-tester or intratester or intra-tester or interobserver or inter-observer or intraobserver or intraobserver or interexaminer or inter-examiner or intraexaminer or intra-examiner or interindividual or inter-individual or intraindividual or intra-individual or kappa or kappa?s or kappas or ((replicab* or repeated) and (measure or measures or findings or result or results or test or tests)) or concordance or (intraclass and correlation*) or (uncertainty and (measurement or measuring)) or "standard error of measurement" or sensitiv*) | 80,499 |
| S14 | AB(valid* or (cronbach* N3 (alpha or alphas)) or interrater or inter-rater or intrarater or intra-rater or intertester or inter-tester or intratester or intra-tester or interobserver or inter-observer or intraobserver or intraobserver or interexaminer or inter-examiner or intraexaminer or intra-examiner or interindividual or inter-individual or intraindividual or intra-individual or kappa or kappa?s or kappas or ((replicab* or repeated) and (measure or measures or findings or result or results or test or tests)) or concordance or (intraclass and correlation*) or (uncertainty and (measurement or measuring)) or "standard error of measurement" or sensitiv*) | 417,988 |
| S13 | (MH "Kappa Statistic") | 17,505 |
| S12 | (MH "Reproducibility of Results") | 67,821 |
| S11 | (MH "Interrater Reliability") | 27,493 |
| S10 | S1 OR S2 OR S3 OR S4 OR S5 | 820,33 |
| S9 | S6 OR S7 OR S8 | 753,761 |
| S8 | TI(clinimetr* or clinometr* or psychometr* or survey? or score or scale or subscale or (measurement N3 instrument) or subscale* or item-discriminant or interscale correlation* or "ceiling effect" or "floor effect" or "Item response model" or Rasch or "Differential item functioning" or "item bank" or (item N3 (correlation* or selection* or reduction* or bank))) | 124,83 |
| S7 | AB(clinimetr* or clinometr* or psychometr* or survey? or score or scale or subscale or (measurement N3 instrument) or subscale* or item-discriminant or interscale correlation* or "ceiling effect" or "floor effect" or "Item response model" or Rasch or "Differential item functioning" or "item bank" or (item N3 (correlation* or selection* or reduction* or bank))) | 694,621 |
| S6 | (MH "Psychometrics") OR (MH "Measurement Issues and Assessments") | 32,107 |
| S5 | AB("Healthcare professionals" or Caregivers or "Healthcare providers" or Practitioners or Doctor or nurse? or physician? or resident? or "healthcare worker" or "health staff") | 556,739 |
| S4 | TI("Healthcare professionals" or Caregivers or "Healthcare providers" or Practitioners or Doctor or nurse? or physician? or resident? or "healthcare worker" or "health staff") | 296,467 |
| S3 | (MM "Nurses+") | 141,674 |
| S2 | (MM "Medical Staff, Hospital+") | 3,977 |
| S1 | (MM "Physicians+") | 64,766 |

**Additional file 2: themes and categories specified**

| **Themes** | **Category** | **Description** |
| --- | --- | --- |
| 1.Management & Building (33) | Workhours (13) | The intervention is about changing shift hours, shift length and schedules |
|  | Continuous improvement (7) | Interventions in which a continuous improvement method is implemented, such as PDCA/Kaizen |
|  | Environment (6) | The intervention is about a change in the space/environment, such as light, sound, room lay-out |
|  | Equipment support & patient handling (4) | The intervention is about a form of support in terms of materials/technology for the purpose of care tasks |
|  | Workflow improvement (2) | Interventions in which the improvement of steps and processes and the reorganization of healthcare are central |
|  | Care model (1) | The interventions is about a framework or system that outlines the principles, strategies, and practices used to provide healthcare services |
|  |  |  |
| 2. Social resources & support (14) | Emotional support (7) | The intervention is about actions to provide psychological support/empathy/encouragement, such as intervision/messages |
|  | (Additional) staff support (4) | The intervention involves deploying additional personnel, such as supervisors, volunteers |
|  | Optimizing teams (3) | The intervention is about actions in which the team collaboratively works on teamwork. |
|  |  |  |
| 3. Personal development & recovery (6) | Role opportunities (2) | The intervention is about development opportunities within professional roles |
|  | Relax opportunities (2) | The intervention is about facilitating action or periods suitable for relaxation/resting |
|  | Other team/setting opportunities (2) | The intervention describes opportunites to gain experience in a new team/setting |
|  |  |  |
| 4. Multi-categorical (1) | Multi categorical (1) | The intervention contains various categories as described above |


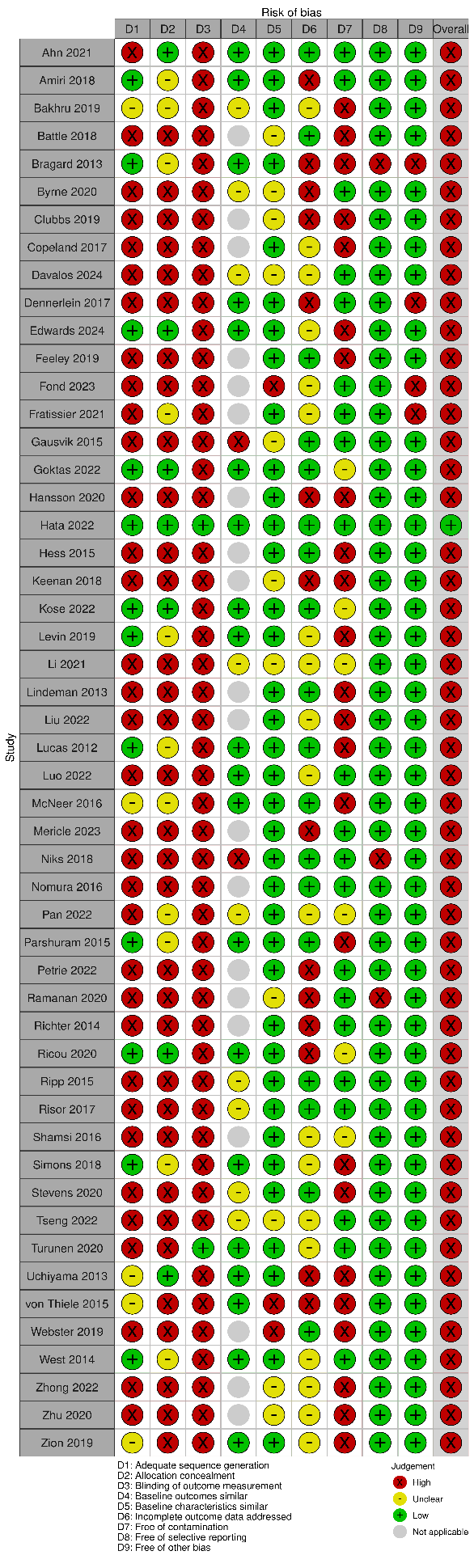
**Additional file 3: risk of bias assessment**

*Additional file 3: Risk of bias assessment -Traffic plot nRCT*


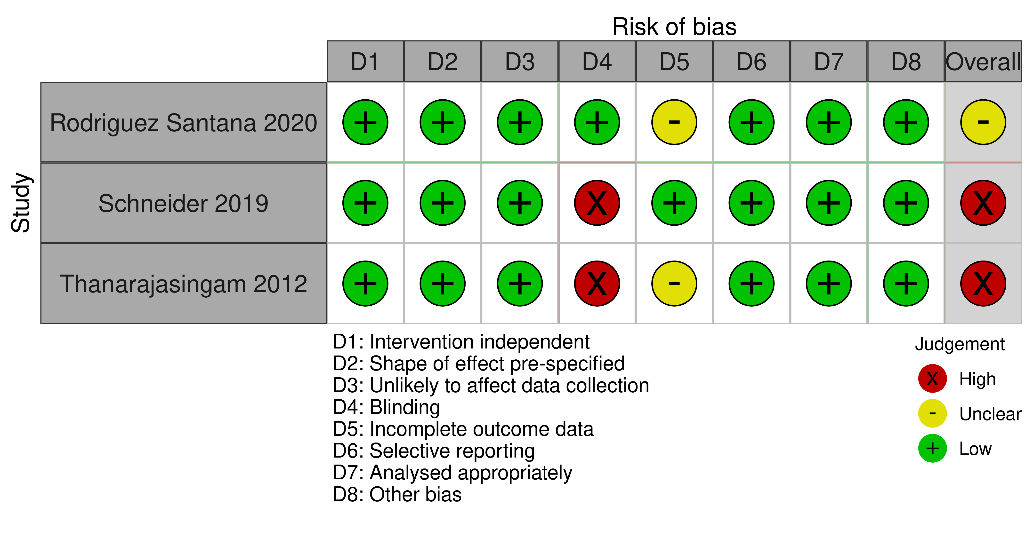


*Additional file 3: Risk of bias assessment -Traffic plot ITS*


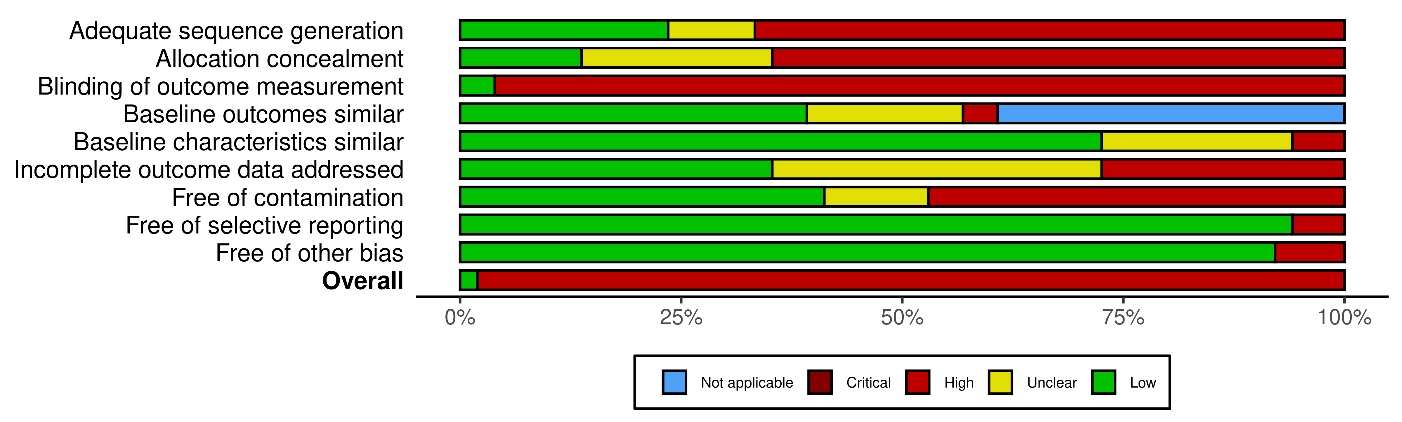


*Additional file 3: Risk of bias assessment -Summary plot nRCT*


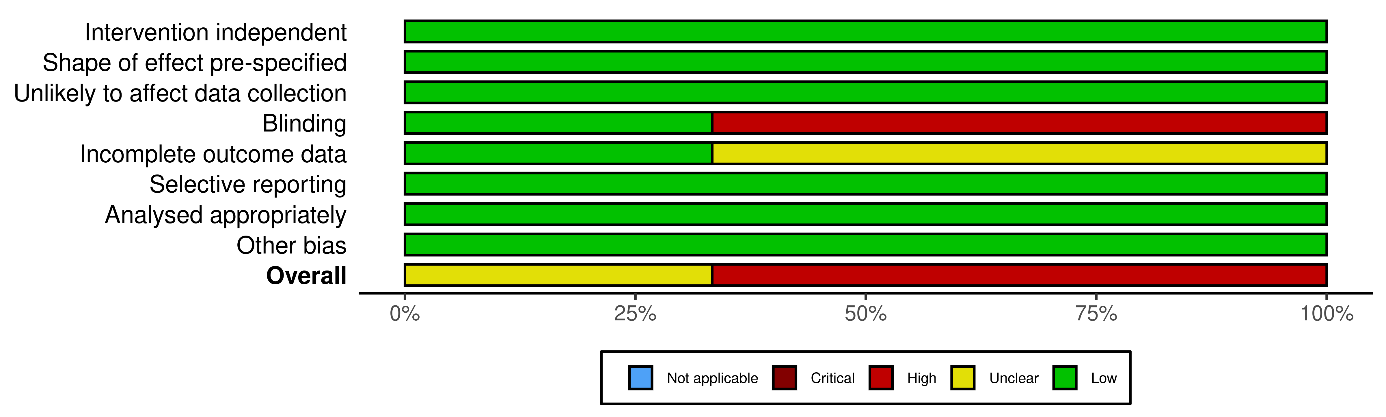


*Additional file 3: Risk of bias assessment - Summary plot ITS*

**Additional file 4: GRADE tables**

The GRADE methodology has been applied to show the strength of evidence for a particular intervention and how reliable the conclusions can be drawn. We included studies and interventions in the GRADE table that (1) evaluate an outcome deemed critical by the WHO, and (2) are included in the forest plots because the reporting of these studies is clearer and therefore more assessable. The GRADE tables are organized by intervention type (management & building, social resources & support, personal development & recovery, and multi-categorical). We did this because other divisions in the analysis, such as by intervention category and HCP, have already been made. This allows data to be presented at different levels. We have described the impact and certainty for the outcomes of workload, burnout, depression, job satisfaction, employability, and patient safety. We made a distinction between evidence from RCTs and non-RCTs. Inconsistency was assessed using the effect direction table. Indirectness was rated as not serious since all populations, interventions, comparisons and outcomes were within the broad scope of our research question. Due to a lack of pooled results, we considered significance and sample size, with a sample size cutoff of 400, for imprecision. The impact is described narratively, also based on the effect direction table. For transparency and readability, a description of the interventions has been provided here. All evidence and certainty have been assessed as very low or low.

**Management & building
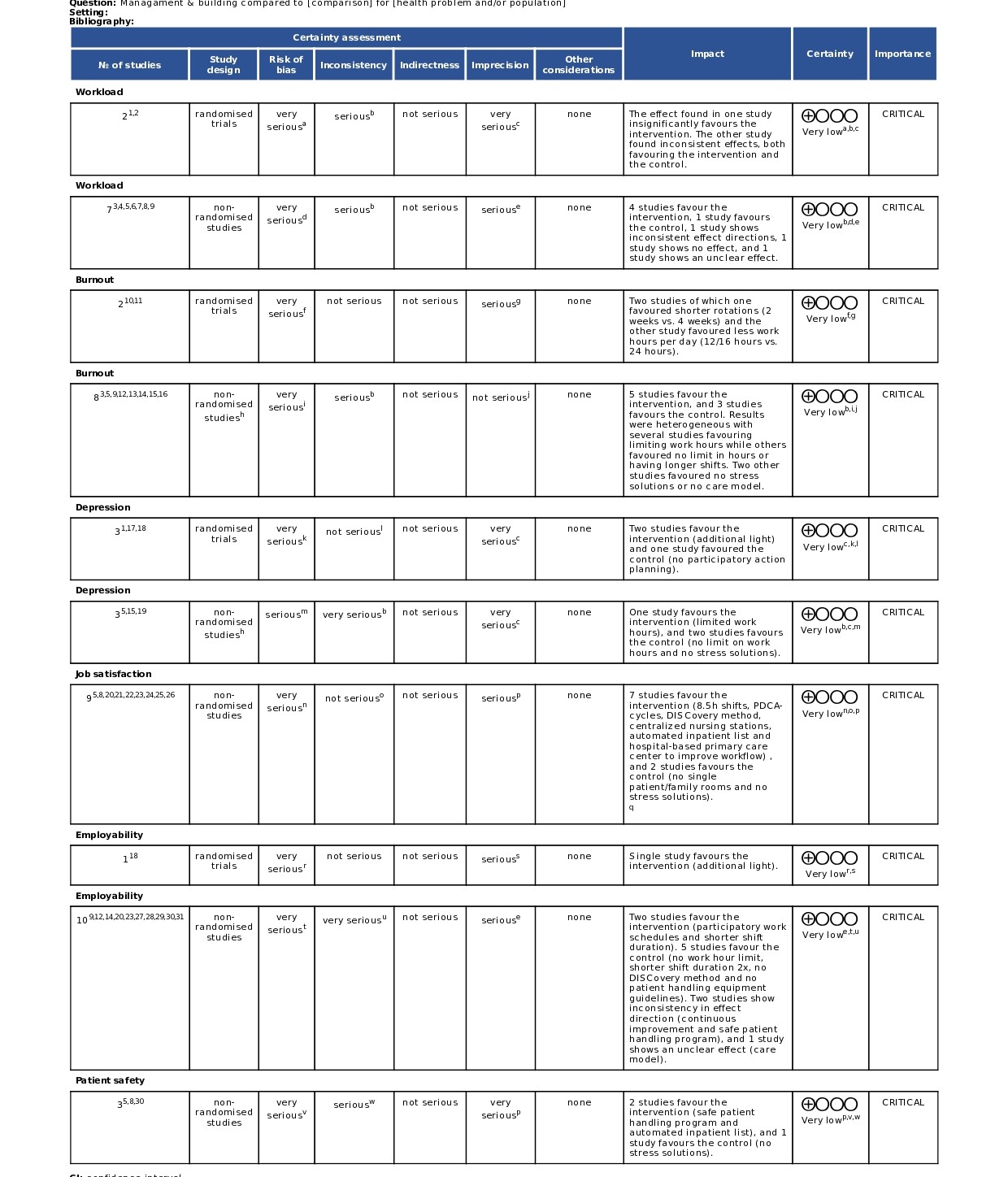
**

Explanations (a) One RCT and one randomized crossover study at high risk of bias. Both have unclear sequence generation, lack of blinding of the outcome assessment and had risk of contamination. (b) Different effect directions of studies. (c) Nonsignificant results and sample size below 400. (d) All studies of high risk of bias, due to lack of blinding in all studies, incomplete outcome data in two studies, risk of contamination in one study. domains were unclear. Five studies had one or more unclear domains. (e) Overall sample size is over 400, however most studies show insignificant results or significance of results is unknown. (f) Both studies at high risk of bias due to unclear allocation concealment, a lack of blinding and risk of contamination. (g) Sample size is below 400 and some insignificant results. (h) 1 RCT. (i) All studies had high risk of bias, due to lack of blinding, risk of contamination, noncomparable baseline characteristics, or incomplete outcome data. (j) Sample size is over 400 and 3 out of the 8 studies report non-significant results. (k) High risk of bias due to a lack of blinding and risk of contamination in all studies. One study also had incomplete outcome data, risk of selective reporting and indications for other bias as well. (l) Different effect directions are likely to be explained through the differences in interventions. Two interventions on additional light had the same effect direction. (m) All studies at high risk of bias due to lack of blinding. One study also has a risk of contamination. (n) All studies at high risk of bias, due to lack of blinding, risk of contamination, noncomparable groups at baseline and/or selective outcome reporting. Several studies have unclear risk of bias for several domains. (o) Most studies have the same effect direction. (p) Overall sample size is over 400, almost half shows nonsignificant or unclear significance of effects. (q) Based on the effect direction table. (r) RCT with high risk of bias due to lack of blinding, incomplete outcome data, risk of contamination and selective reporting. (s) Significant effect, but low sample size (n=25). (t) All studies at high risk of bias due to a lack of blinding, incomplete outcome data, risk of contamination, noncomparable groups at baseline, and/or selective outcome reporting. (u) Results regarding the work shift duration seem to point towards the same direction of shorter work hours. Overall the studies show different effect directions. (v) All studies at high risk of bias, due to lack of blinding and incomplete outcome data. One study had unclear comparability on baseline. (w) Two studies on equipment support and handling interventions show similar effect directions, however another study on another intervention showed different effect direction.
 References (1) Uchiyama, . .2013. (2) McNeer, . .2016. (3) Fond, . .2023. (4) Fratissier, . .2021. (5) Schneider, . .2019. (6) Mericle, . .2023. (7) Tseng, . .2022. (8) Davalos, . .2024. (9) Hansson, . .2020. (10) Parshuram, . .2015. (11) Lucas, . .2012. (12) Richter, . .2014. (13) Ripp, . .2015. (14) Battle, . .2018. (15) Lindeman, . .2013. (16) Li, . .2021. (17) Simons, . .2018. (18) Bragard, . .2013. (19) Nomura, . .2016. (20) Webster, . .2019. (21) Luo, . .2022. (22) Pan, . .2022. (23) Niks, . .2018. (24) Feeley, . .2019. (25) Copeland, . .2017. (26) Hess, . .2015. (27) Turunen, . .2020. (28) Rodriguez, . .2020. (29) .Thiele, Von. .2015. (30) Dennerlein, . .2017. (31) Risor, . .2017.

Additional file 4: GRADE table 1 Management & Building interventions

**Social resources & support
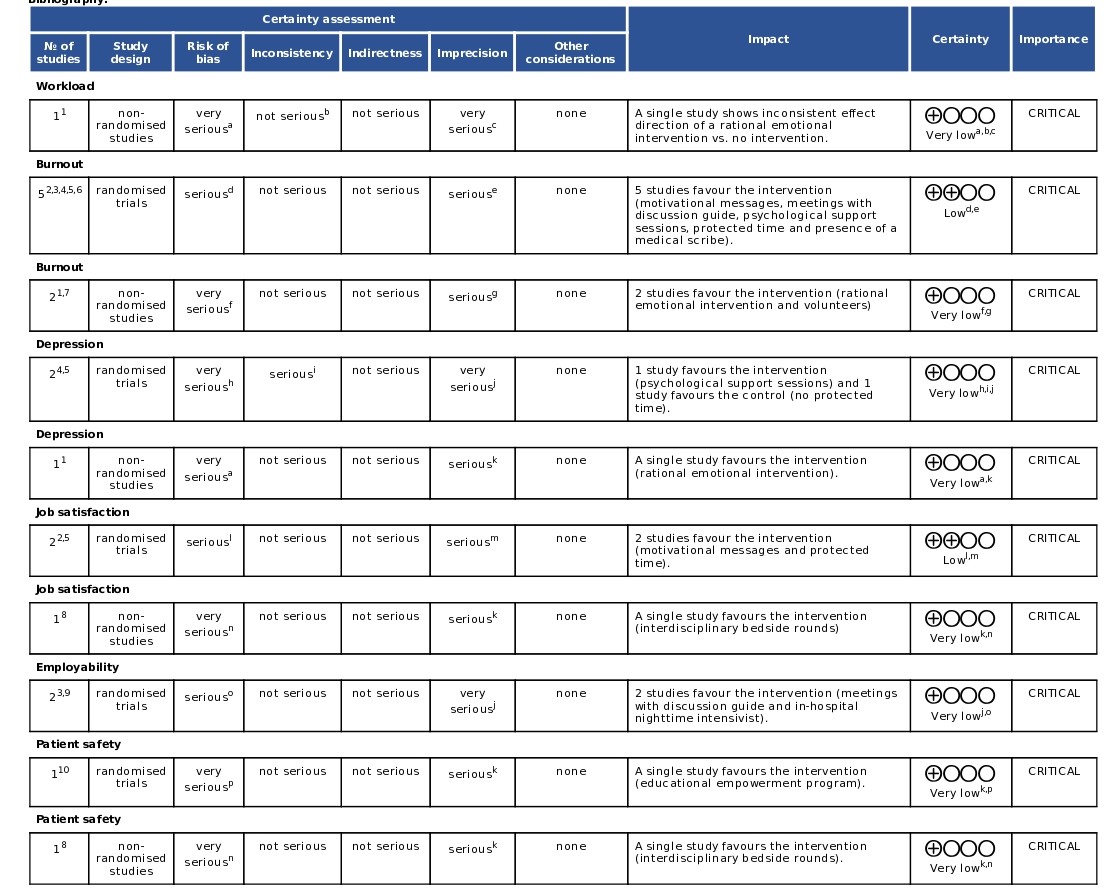
**

Explanations (a) Study at high risk of bias due to lack of blinding, risk of contamination and unclear completeness of outcome data. (b) Single study, so inconsistency not scored. However, there are inconsistent results within study. (c) Sample size below 400 and uncertainty of effect direction. (d) One RCT of low risk of bias and 4 randomized studies at high risk of bias, due to the lack of blinding, incomplete outcome data, and risk of contamination. (e) Sample size is over 400, however three out of five studies show insignificant or unclear significance of results. (f) Both studies at high risk of bias, due to lack of blinding, incomplete outcome data and/or risk of contamination. (g) Sample size below 400 and both show significant results. (h) Both studies at high risk of bias, due to unclear allocation concealment, lack of blinding, incomplete or unclear completeness of outcome data and unclear risk of contamination. (i) Different effect directions of studies. (j) Sample size below 400 and insignificant results. (k) Sample size below 400, but significant results. (l) Both studies at high risk of bias due to the lack of blinding. One study has unclear allocation concealment and one study has unclear risk of contamination. (m) Sample size over 400, with one study having insignificant results. (n). Study at high risk of bias due to lack of blinding and noncomparable groups at baseline. (o) One study with low risk of bias and one study with high risk of bias due to unclear sequence generation and allocation concealment, a lack of blinding and risk of contamination. (p) Study at high risk of bias due to unclear allocation concealment, lack of blinding, risk of contamination and unclear completeness of data.
 References (1) 1.Liu, . .2022. (2) Goktas, . .2022. (3) Hata, . .2022. (4) Ricou, . .2020. (5) West, . .2014. (6) Edwards, . .2024. (7) Clubbs, . .2019. (8) Gausvik, . .2015. (9) .Bakhru, . .2019. (10) Amiri, . .2018.

Additional file 4: GRADE table 2 Social resources & support interventions


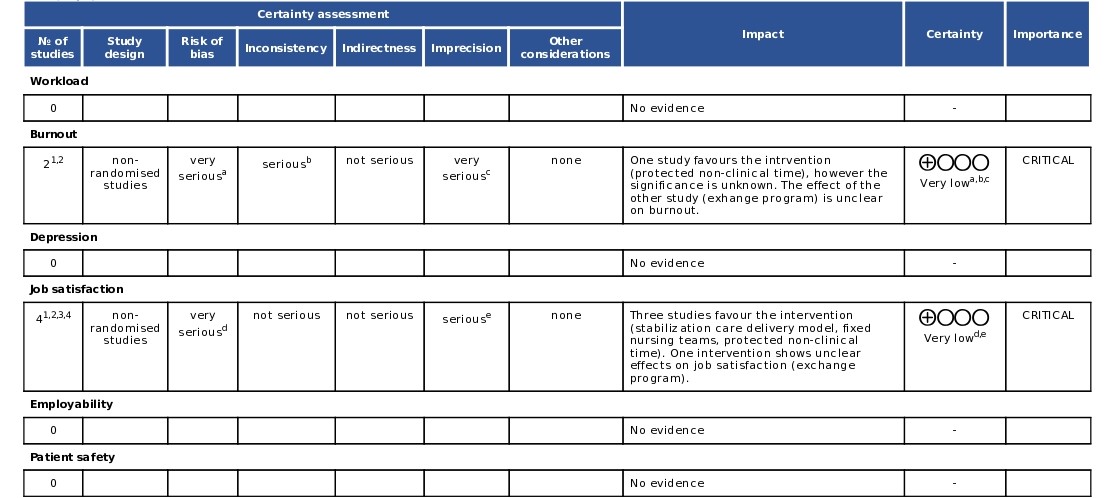
 **Personal development & recovery**

Explanations (a) All studies of high risk of bias, due to lack of adequate sequence generation, allocation concealment, and blinding. Baseline outcomes were unclear for both studies. Baseline characteristics were unclear for one study. One study shows incomplete outcome data and one study shows high risk on contamination. (b) One study favours intervention, the other study shows an unclear effect. (c) Nonsignificant results and sample size below 400. (d) All studies of high risk of bias, due to lack of adequate sequence generation, allocation concealment, blinding, contamination, and incomplete outcome data. All studies had one or more unclear domains. (e) Overall sample size is over 400, however one study show unknown significance and one study unclear effects.
 References (1) Stevens, . .2020. (2) Byrne, . .2020. (3) Shamsi, . .2016. (4) Zhong, . .2022.

Additional file 4: GRADE table 3 Personal development & recovery interventions

**Multi-categorical**


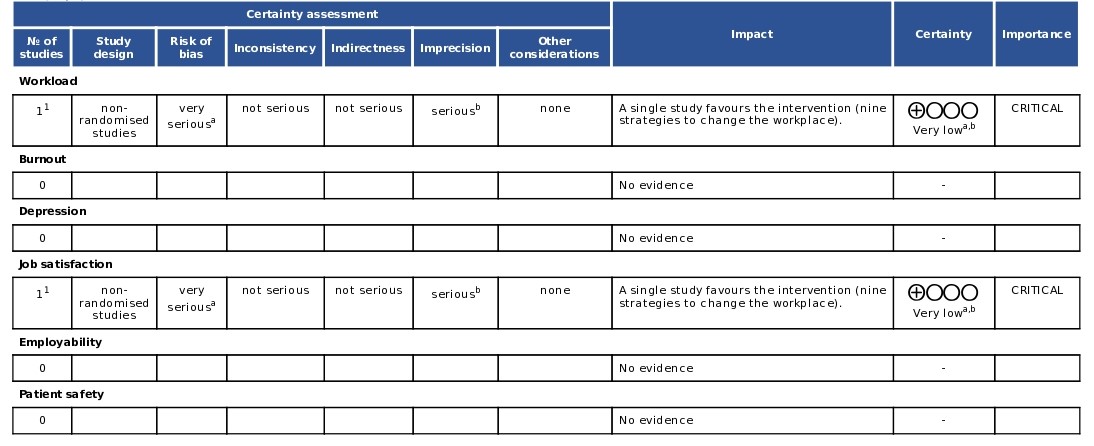


Explanations (a) High risk of bias, due to lack of adequate sequence generation, allocation concealment, blinding, and incomplete outcomes. (b) Significant results, however sample size is below 400.
 References (1) Petrie, . .2022.

Additional file 4: GRADE table 4 Multi-categorical intervention

**Additional file 5: Forest plots**

Feasibility of meta-analyses was assessed, and forest plots with standardized mean differences (SMDs) were created for continuous variables. Due to heterogeneity, no pooled effect is presented. The plots should be interpreted with caution due to heterogeneity. To account for heterogeneity, studies are presented within intervention categories, and as much information as possible is included in plots, such as intervention/comparator and measurement instrument. Meta-analysis was only performed on an exploratory basis.


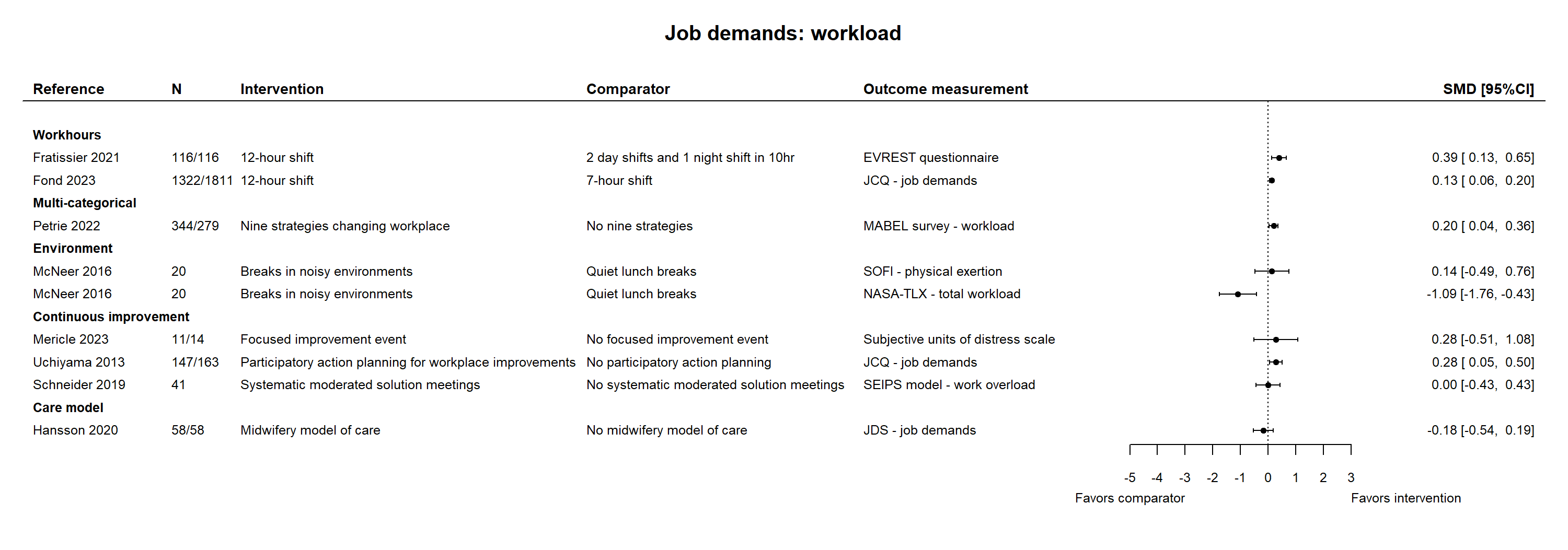


Notes: MABEL: Medicine in Australia: Balancing employment and life; SOFI: Swedish occupational fatigue inventory; NASA-TLX: National Aeronautics and Space Administration - Task Load Index; JCQ: Job content questionnaire; SEIPS: Systems engineering initiative for patient safety; JDS: Job demands scale

*Additional file 5: Forest plot A – Job demands: Workload*


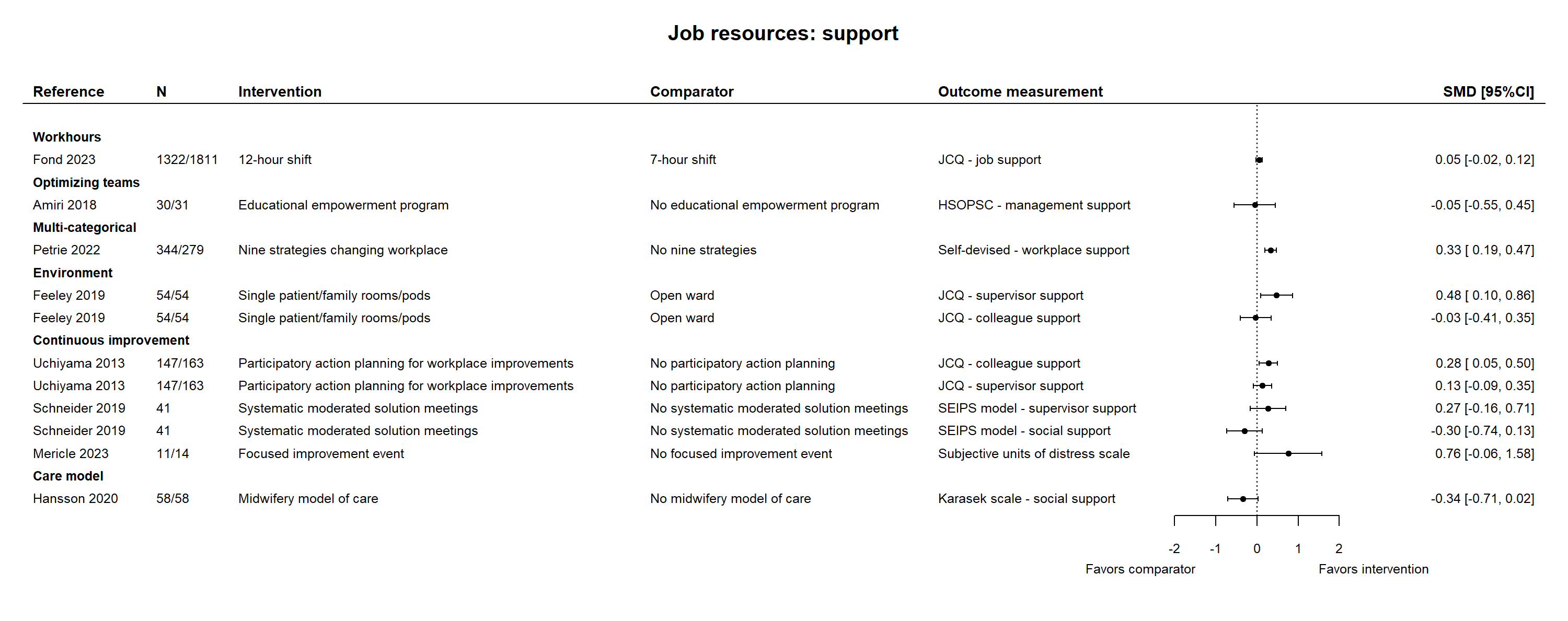


Notes: HSOPSC: Hospital survey on patient safety culture; JCQ: Job content questionnaire; SEIPS: Systems engineering initiative for patient safety

*Additional file 5: Forest plot B – Job resources: Support*

*
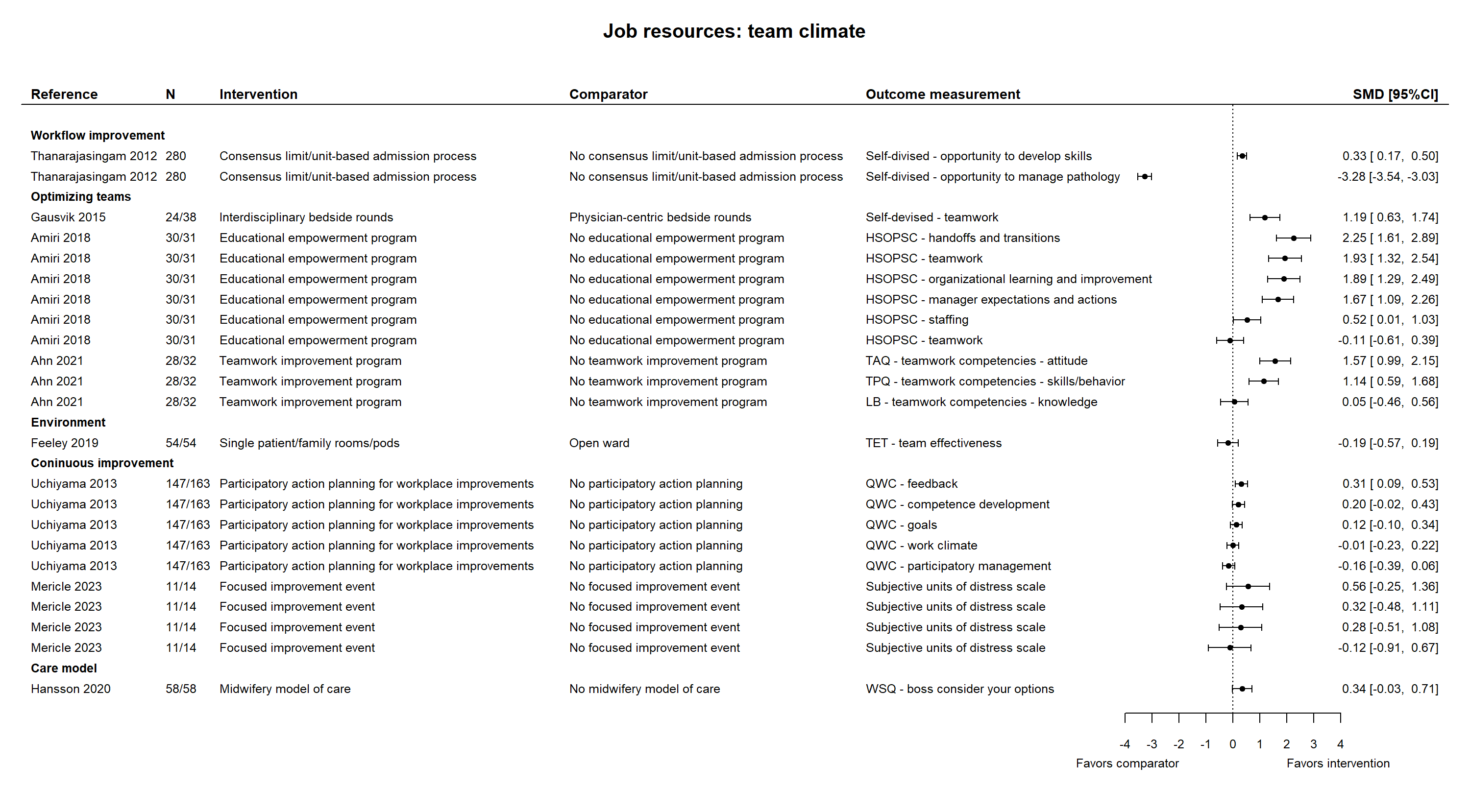
* Notes: HSOPSC: Hospital survey on patient safety culture; TAQ: Teamwork attitudes questionnaire; TPQ: Teamwork perceptions questionnaire; LB: Learning benchmarks; TET: Team effectiveness tool: QWC: Quality work competence questionnaire; WSQ: Work stress questionnaire

*Additional file 5: Forest plot C – Job resources: Team climate*


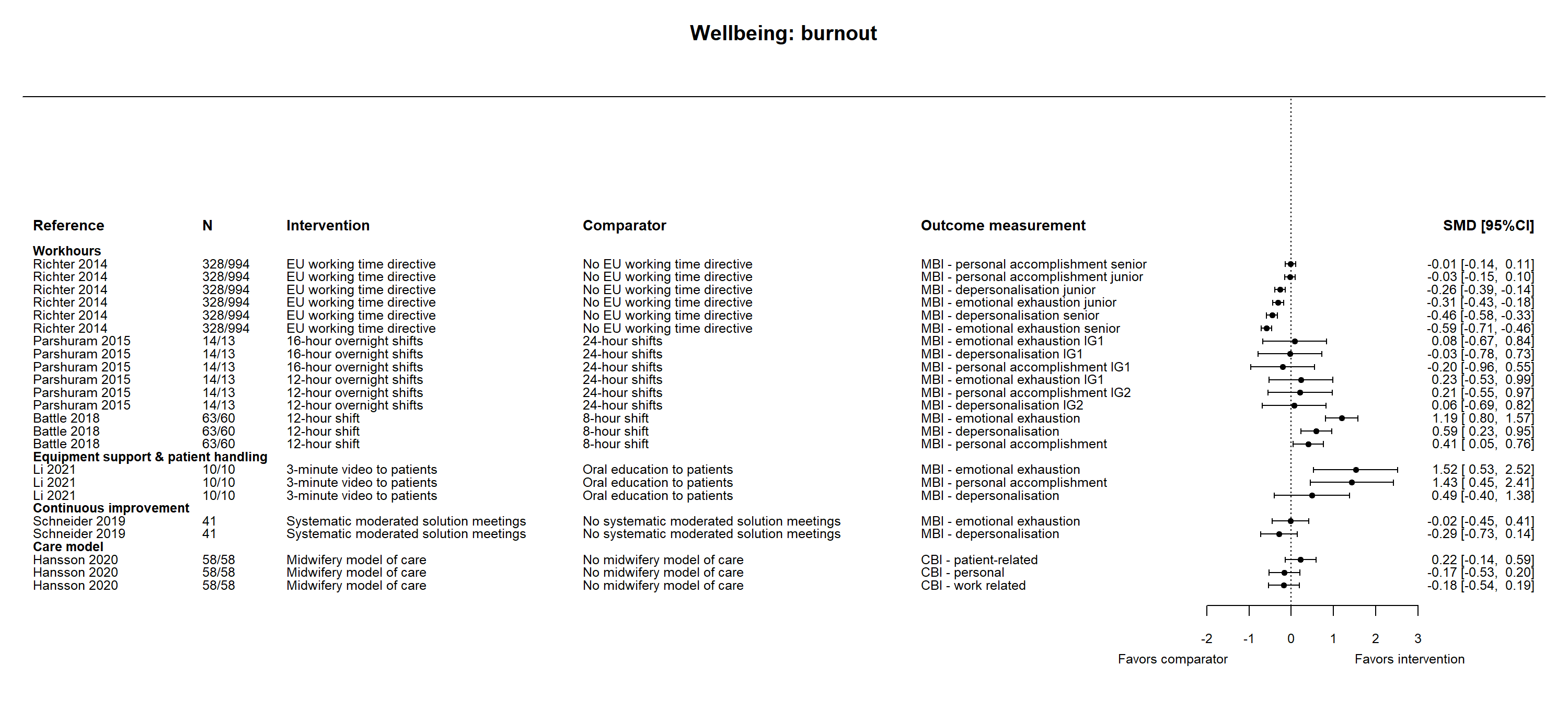


Notes: MBI: Maslach burnout inventory; CBI: Copenhagen burnout inventory

*Additional file 5: Forest plot D – Wellbeing: Burnout*


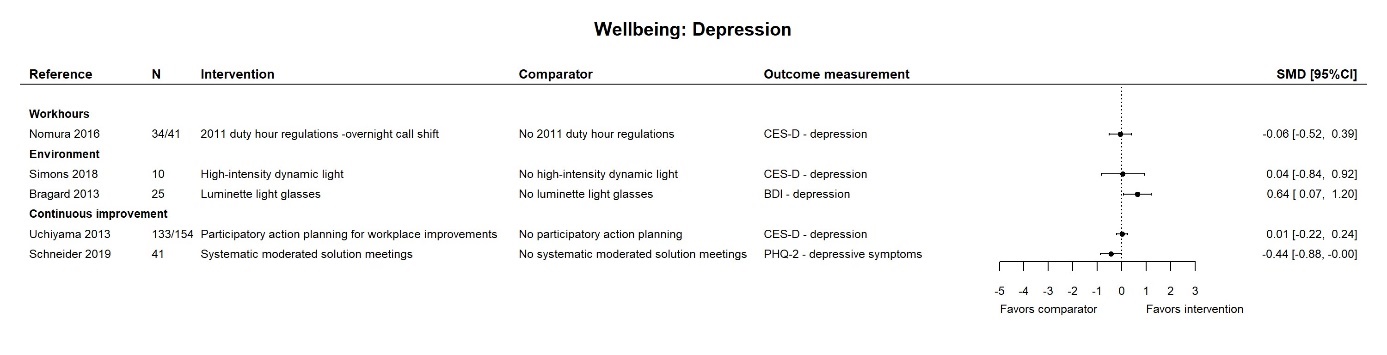


Notes: CES-D: Center for epidemiologic studies depression scale; BDI: Beck depression scale; PHQ-2: Patient health questionnaire 2

*Additional file 5: Forest plot E – Wellbeing: Depression*


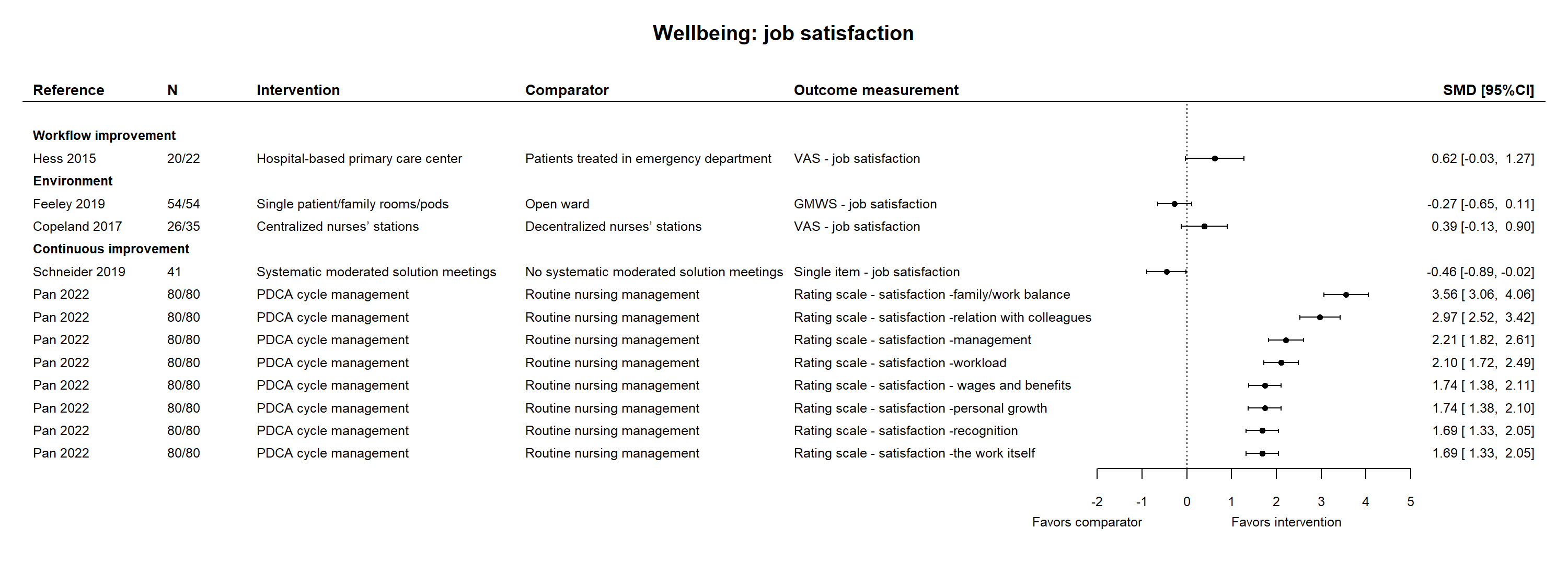
 Notes: VAS: Visual analog scale; GMWS: Global measure of work satisfaction

*Additional file 5: Forest plot F – Wellbeing: Job satisfaction*

Notes: SF-36: Short form 36; UWES: Utrecht work engagement scale; HWQ: Health and work questionnaire; WAI: Work ability index; VAS: Visual analog scale
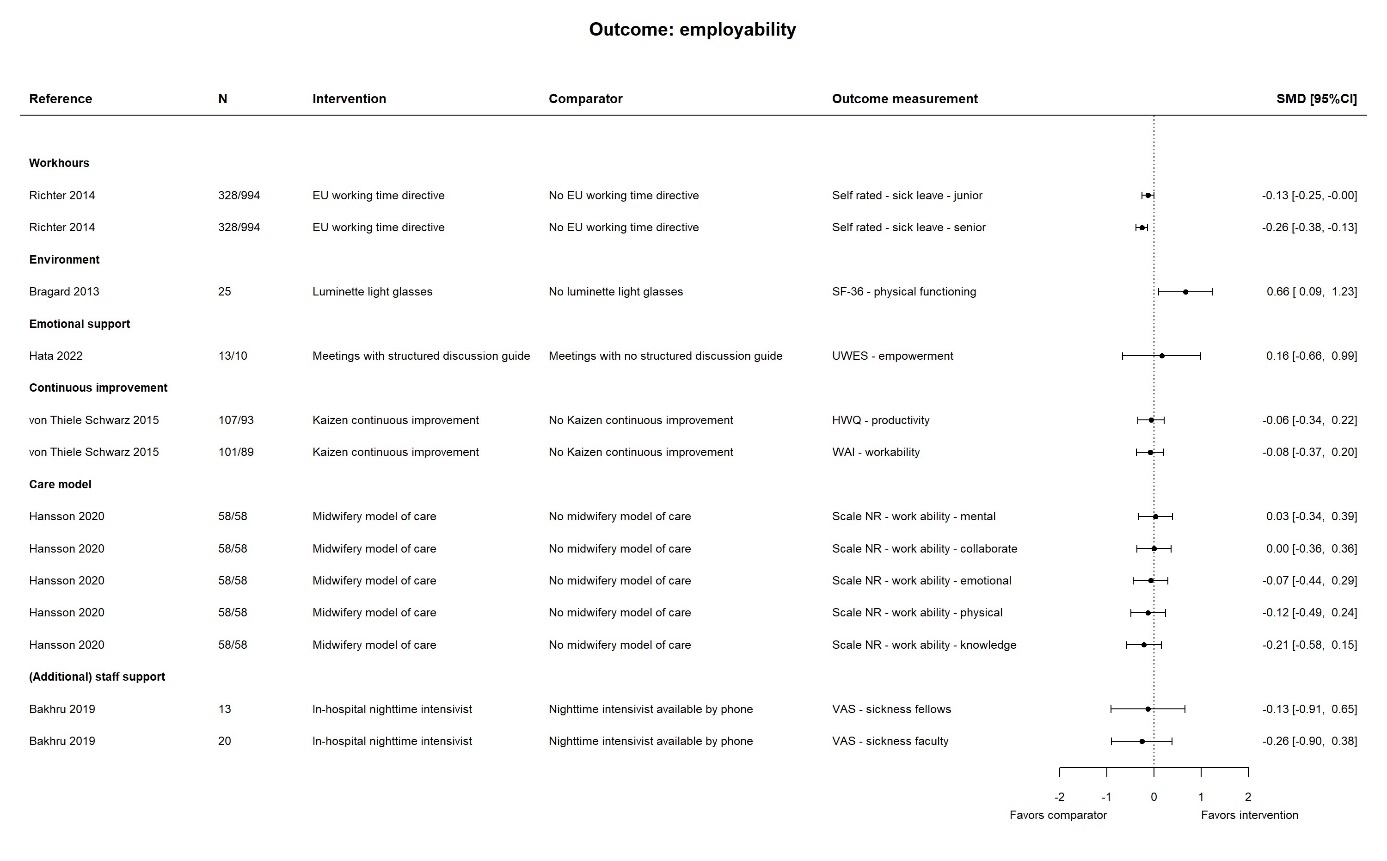


*Additional file 5: Forest plot G – Outcome: Employability*


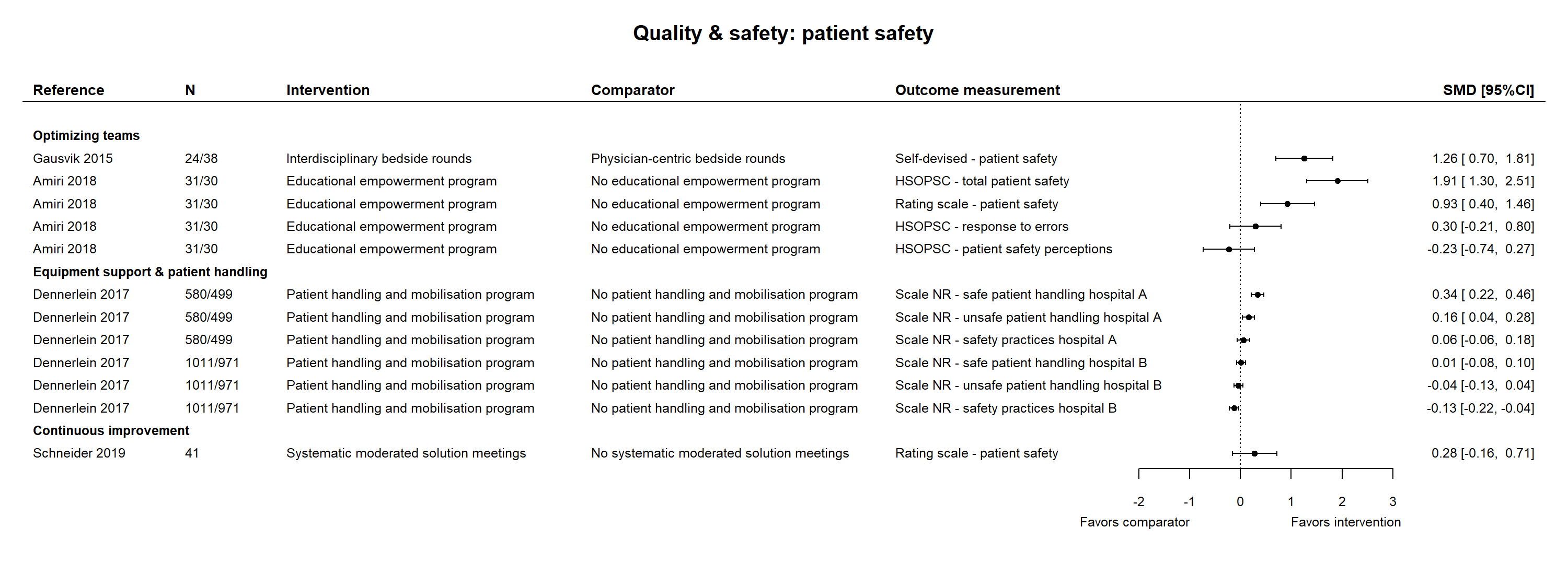
 Notes: HSOPSC: Hospital survey on patient safety culture; NR: Not reported

*Additional file 5: Forest plot H – Quality & safety: Patient safety*

**Additional file 6: Direction table complete**

**
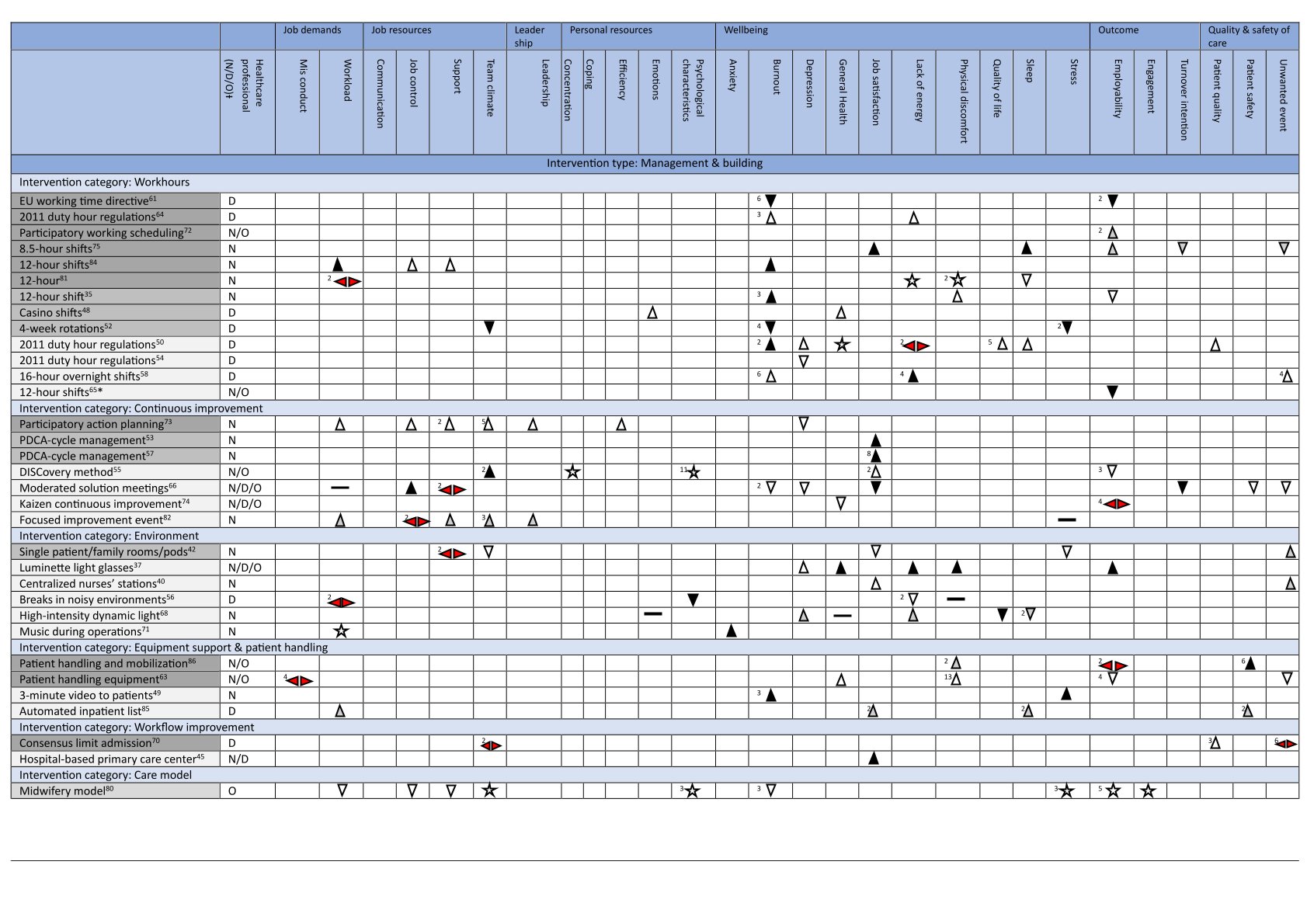
**


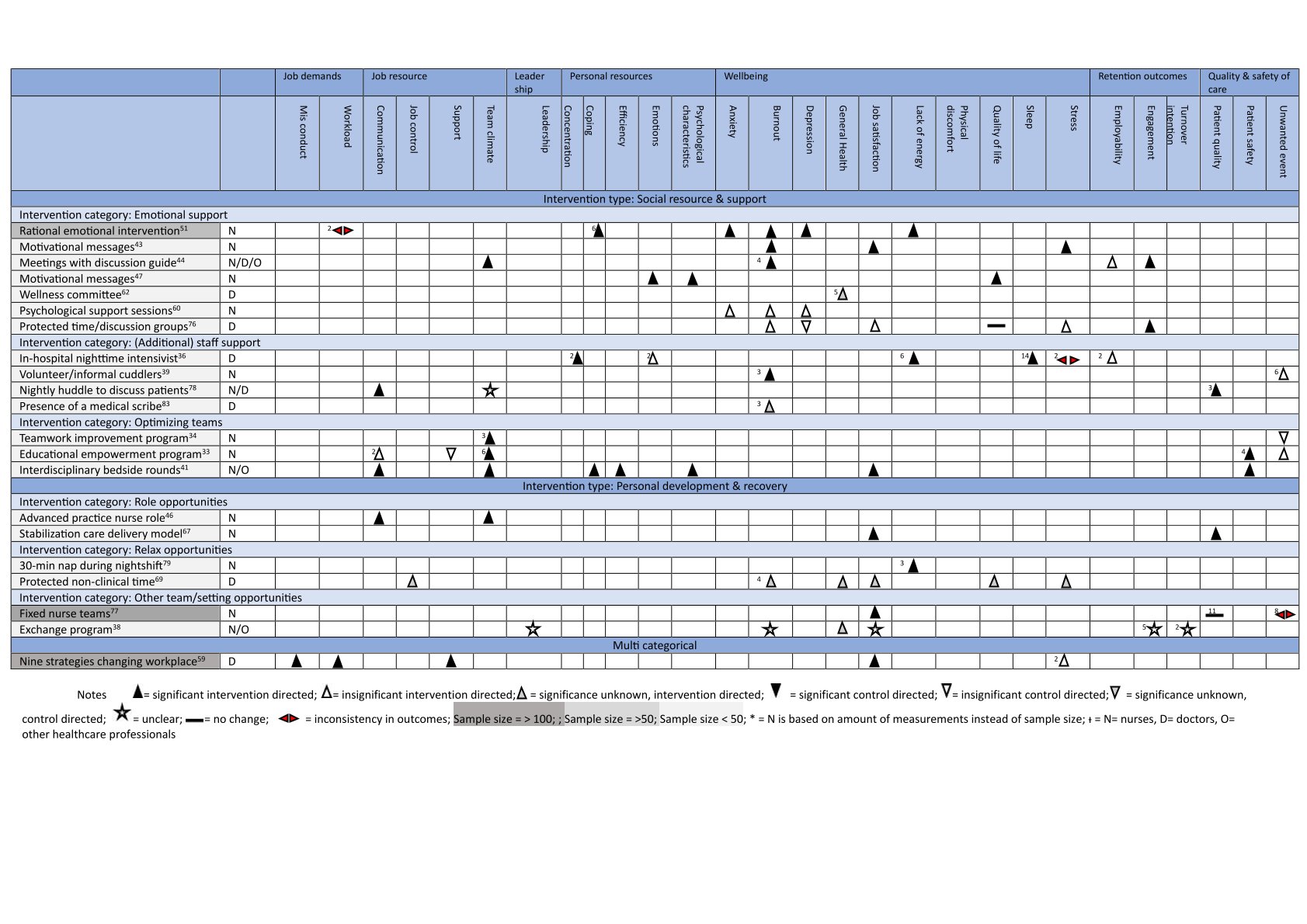


**Additional file 7: Direction table nurses**

**
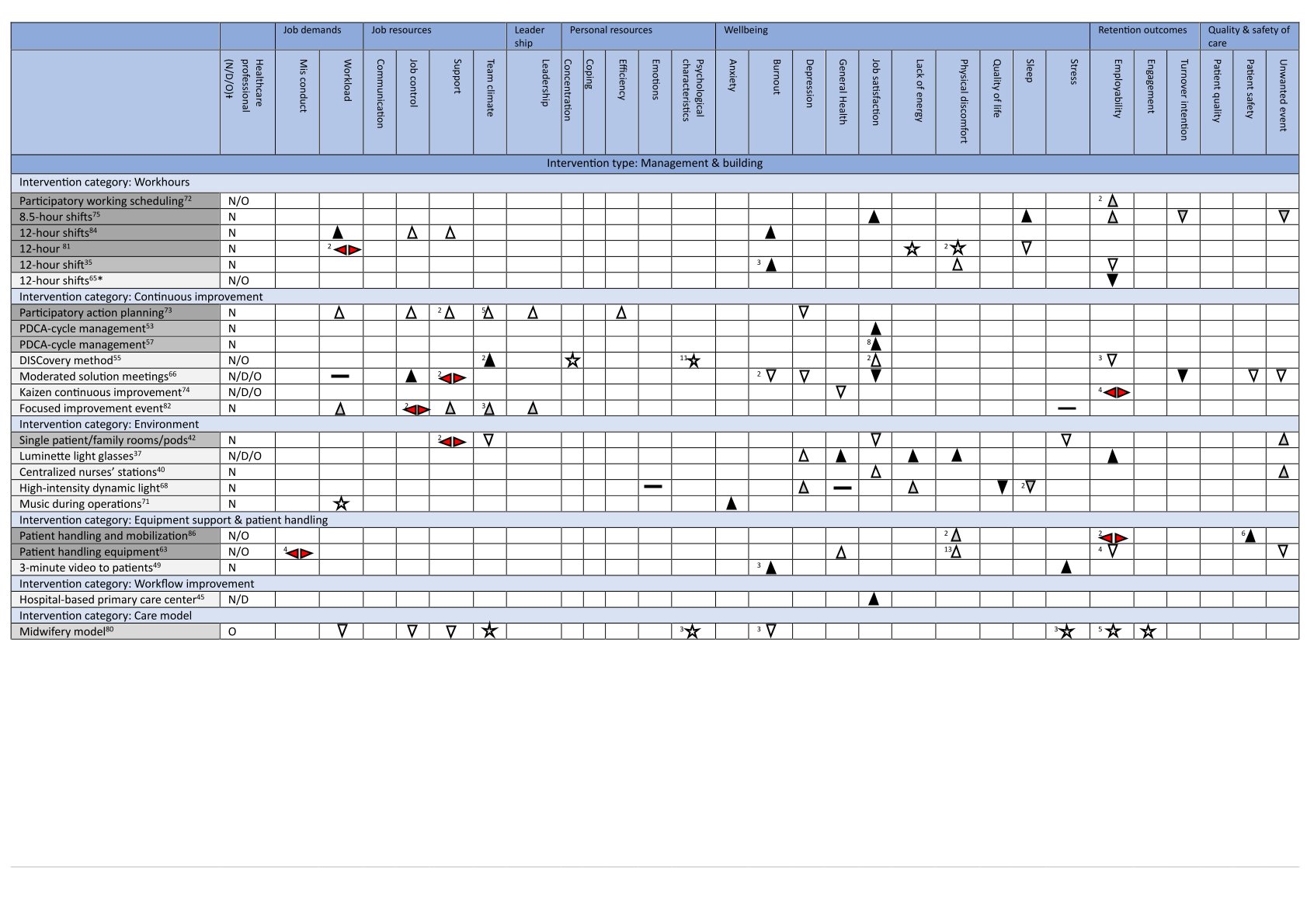
**


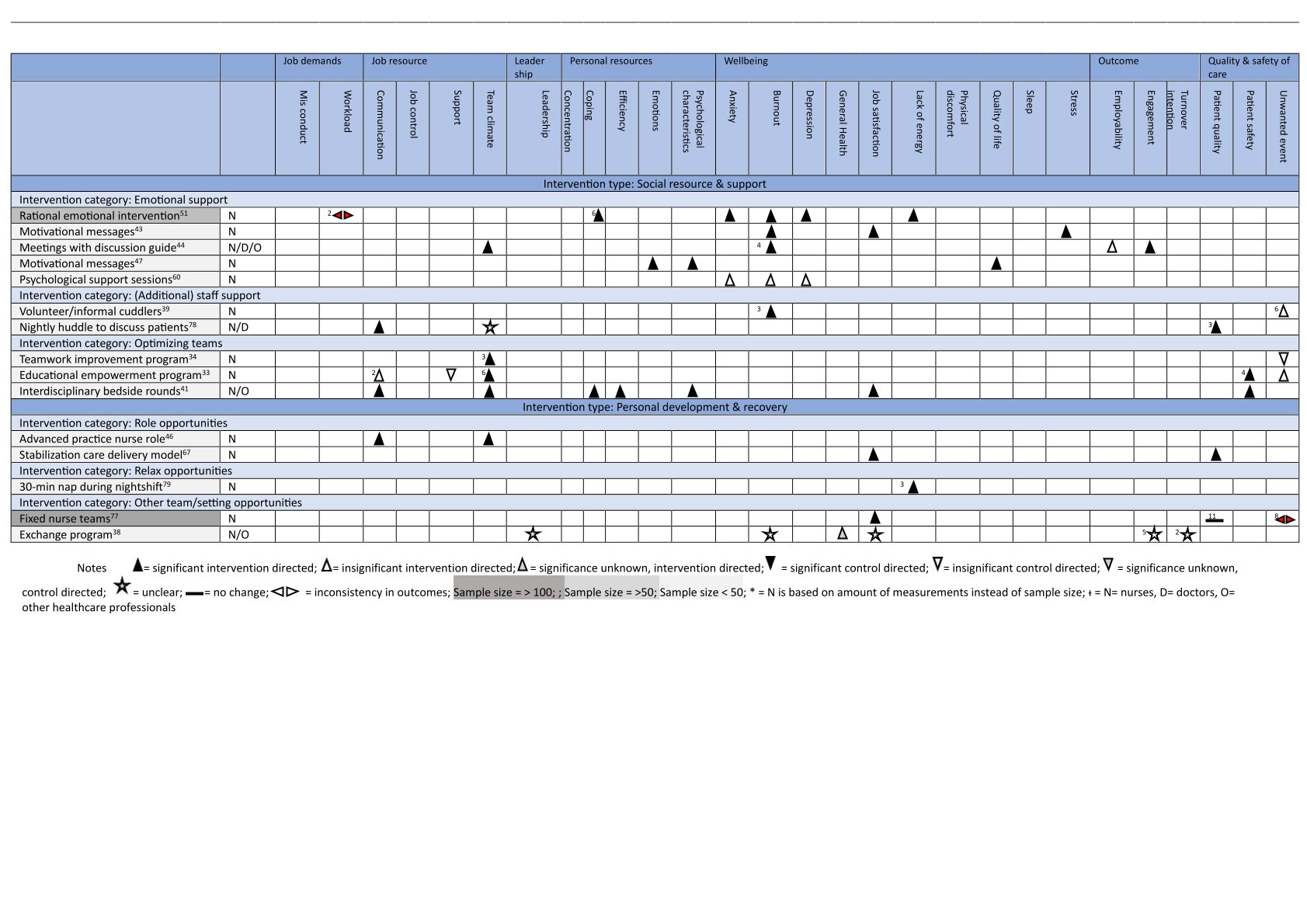


**Additional file 8: Direction table doctors**


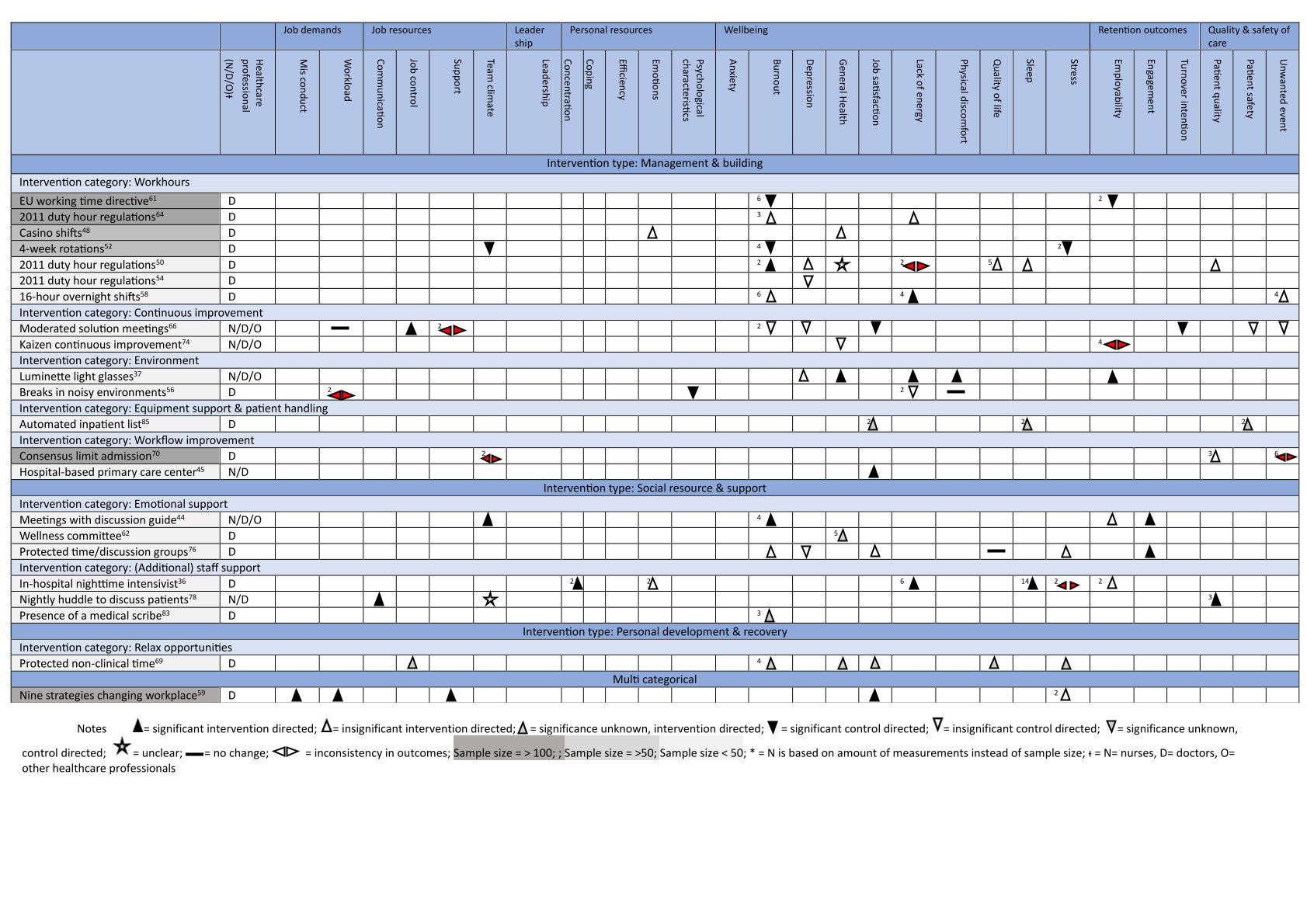


**Additional file 9: Description of results on outcomes Job resources, Leadership and Personal resources**

Job demands

*Misconduct*Two studies measured misconduct outcomes59,63, assessing experienced bullying, aggressiveness and/or harassment. One significant favored a multi-categorical intervention59, which used nine strategies to reduce un-rostered overtime and enhance doctors’ well-being.

Job resources

*Communication*
Four studies measured communication outcomes33,41,46,78. One used a subscale of the Hospital survey on patient safety culture; three used self-developed questionnaires. Three significant favored the intervention78,41,46, including ‘staff support’ (nightly huddle to discuss patients), ‘optimizing teams’ (interdisciplinary versus physician bedside rounds), and ‘role opportunities’ (advanced practice nurse).

*Job control*
Six studies measured job control outcomes66,69,73,80,82,84, using four questionnaires (e.g., Mini-Z survey). One significant favored a ‘continuous improvement’ intervention66, with professional solution meetings versus no meetings.

*Support*
Eight studies measured experienced support outcomes33,42,59,66,73,80,82,84, using six questionnaires (e.g., SEIPS model, job content questionnaire). One significant favored a ‘multi-categorical’ intervention versus no intervention59. All eight studies were appropriate for meta-analysis. Given the variation in effect direction but overlapping confidence intervals within interventions categories, the effects on support show moderate heterogeneity with SMDs from -0.34 to 0.76.

*Team climate*
Thirteen studies measured team climate33,34,41,42,44,46,52,55,70,73,78,80,82, using eleven questionnaires (e.g., Work stress questionnaire). Six significant favored the interventions33,34,41,46,55: one ‘continuous improvement’ (DISCovery Method versus no intervention), one ‘emotional support’ (monthly self-facilitated group meetings with/without structured discussion guide), three ‘optimizing teams’ (e.g., two learning programs for teams versus no intervention), and one ‘role opportunities’ (advanced practice nurse versus no intervention). Lucas et al. found the control (2-week rotations) more effective than the intervention (4-week rotations). Eight studies were appropriate for meta-analysis (plot C)33,34,41,42,70,73,80,82, showing heterogeneity with variation in effect direction and non-overlapping confidence intervals within intervention categories, with SMDs from -3.28 to 2.25. The SMD of -3.28 can be described as an outlier, as this data point markedly deviates from the rest. ‘Optimizing teams’ and ‘equipment support & patient handling’ interventions showed most potential for improving team climate.

Leadership
Three studies measured leadership outcomes38,73,82, using three instruments (e.g., Self rated leadership). None significant favored the intervention.

Personal resources

*Concentration*Two studies measured concentration outcomes36,55, using two questionnaires for self-rated concentration/alertness/focus problems (e.g., visual analog scale). One indicated significant effects: one favoring a ‘staff support’ intervention (nighttime staffing in hospital versus phone ability)36.

*Coping*
Two studies measured coping outcomes41,51, using two questionnaires for response/reaction to uncertainty/stress/problems/fear (e.g., Coping style questionnaire). Both indicated significant effects: one for an ‘emotional support’ intervention (hierarchical management versus no intervention)51, and one for an ‘optimizing teams’ intervention (interdisciplinary versus physician bedside rounds)41.

*Efficiency*
Two studies measured efficiency outcomes41,73, using two questionnaires of which one was self-developed. One significant favored an ‘optimizing teams’ intervention (interdisciplinary versus physician bedside rounds)41.

*Emotions*
Four studies measured emotions outcomes36,47,48,68, using three questionnaires for self-rated mood/happiness/hopelessness/positivity (e.g., Brief resident wellness profile). One significant favored an ‘emotional support’ intervention (breaks with/without motivational messages)47.

*Psychological characteristics*
Five studies measured psychological characteristics outcomes41,47,55,56,80, using five questionnaires, e.g., on motivation/responsibility (e.g., Life orientation test). Two significant favored ‘emotional support’ interventions (breaks with versus without motivational messages)47, and an ‘optimizing teams’ intervention (interdisciplinary versus physician bedside rounds)41. McNeer et al. showed significant effects favoring the control group (quite lunchbreaks) over the intervention (noisy lunchbreaks)56.

**Additional file 10: An overview of screened full-text articles excluded with corresponding reasons**

| **Search** | **Title** | **Author** | **Reason** |
| --- | --- | --- | --- |
| First search | Nurse-Physician Teamwork in the Emergency Department | Ajeigbe | Wrong design |
| Update | Quality of care and job satisfaction in a Hospital Trust before | Andersen | Wrong/no intervention |
| First search | and after The Coordination Reform in Norway | Armada | Wrong design |
| First search | Comparative Analysis of Emergency Medical Service Provider Workload During Simulated Out-of-Hospital Cardiac Arrest Resuscitation Using Standard Versus Experimental Protocols and Equipment | Asselin | Wrong population |
| First search | Better rested, but more stressed? Evidence of the effects of resident work hour restrictions | Auger | Wrong outcome |
| Update | Application of the PDCA Cycle for Nursing Safety Management in | Bai | Wrong outcome |
| First search | Effectiveness of the computerization of a YALE insulin infusion modified protocol in reducing the nursing workload in an Internal Medicine Department | Beltramello | Wrong population |
| First search | Effectiveness of a 'do not interrupt' vest intervention to reduce medication errors during medication administration: a multicenter cluster randomized controlled trial | Berdot | Wrong outcome |
| First search | Impact of a pharmacist-run refill and prior authorization program on physician workload | Bhakta | Wrong population |
| First search | Development of the Flexibility in Duty Hour Requirements for Surgical Trainees (FIRST) Trial Protocol: A National Cluster-Randomized Trial of Resident Duty Hour Policies | Bilimoria | Wrong population |
| First search | The impact of rest breaks on subjective fatigue in physicians of the General Hospital of Vienna | Blasche | Wrong design |
| First search | Influence of ability level principle based on human resource management on ward nursing quality and satisfaction in surgical nursing management | Cao | Wrong/no intervention |
| First search | Physical and Mental Impact of Laparoscopic Sleeve Gastrectomy on the Surgeon: French vs. American Positions. A Randomized and Controlled Study | Carmona | Wrong/no intervention |
| First search | Comparison of Shift Satisfaction, Sleep, Fatigue, Quality of Life, and Patient Safety Incidents Between Two-Shift and Three-Shift Intensive Care Unit Nurses | Chae | Wrong design |
| Update | Effectiveness of transition programs on new graduate nurses’ clinical competence, job satisfaction and perceptions of support: A mixed-methods study | Charette | Wrong/no intervention |
| Update | Acuity-based rotational patient-to-physician assignment in an emergency department using electronic health records in triage | Cildoz | Wrong outcome |
| First search | Achieving balance on the inpatient internal medicine wards: a performance improvement project to restructure resident work hours at a tertiary care center | Cohee | No full-text in English |
| First search | Outcomes of adding acute care nurse practitioners to a Level I trauma service with the goal of decreased length of stay and improved physician and nursing satisfaction | Collins | Wrong design |
| First search | Electronic Staff Unit Meetings: A New Model | Courtney | Wrong outcome |
| First search | Changing nurses' views of the therapeutic environment: randomized controlled trial | Csipke | Wrong/no intervention |
| First search | Addressing Provider Burnout, Improving Provider Satisfaction, and Increasing Patient Access through Nurse Practitioner-Physician Primary Care Teams | D'Afflitti | Wrong population |
| Update | Safer anesthetic rooms: Human factors/ergonomics analysis of work practices | Davis | Wrong/no intervention |
| Update | An examination of the usefulness of a quantitative appraisal method in nursing human resource management in primary hospital operating rooms An example of integrated collaborative scheduling | Ding | Wrong/no intervention |
| First search | A protocol to measure the impact of intentional changes to nurse staffing and skill-mix in medical and surgical wards | Drennan | Wrong publication type |
| First search | Burnout in the emergency department: Randomized controlled trial of an attention-based training program | Dunne | Wrong/no intervention |
| First search | Effects of a Multimodal Program Including Simulation on Job Strain Among Nurses Working in Intensive Care Units: A Randomized Clinical Trial | El Khamali | Wrong/no intervention |
| Update | Effect of Job Crafting Intervention Program on Harmonious Work Passion and Career Commitment among Nurses: A Randomized Controlled Trial | El-Gazar | Wrong/no intervention |
| First search | Impact of implementation of the National Early Warning Score on patients and staff | Farenden | Wrong outcome |
| First search | Two's Company'. Transitioning from One to Two on-Call Registrars in an Irish Paediatric Tertiary Hospital, the Non-Consultant Hospital Doctor's Perspective | Fitzgerald | Wrong publication type |
| Update | Evaluating associations between patient-to-nurse- ratios and mortality, process of care events and vital sign documentation on pediatric wards: a secondary analysis of data from the EPOCH cluster-randomized trial | Gawronski | Wrong/no intervention |
| First search | Changes in surgical team performance and safety climate attitudes following expansion of perioperative services: a repeated-measures study | Gillespie | Wrong/no intervention |
| First search | Brainstorming Our Way to Improved Quality, Safety, and Resident Wellness in a Resource-Limited Emergency Department | Ginocchio | Wrong outcome |
| First search | Effectiveness of an intervention for prevention and treatment of burnout in primary health care professionals | Gomez-Gascon | Wrong publication type |
| Update | Influence of Group Training Based on Psychological Capital Theory on Nursing Staff’s Occupational Benefits and Job Satisfaction in an Infusion Preparation Center | Gon | Wrong/no intervention |
| First search | The impact of nursing rounds on the practice environment andnurse satisfaction in neuroscience intensive care unit | Gu | Wrong publication type |
| First search | Improving Wellness of Operating Room Personnel: A Light-Based Intervention on Perceived Nursing-Related Stress | Guerrier | Wrong/no intervention |
| First search | Impact of Dispense Tracking Software on Inpatient Pharmacy Operations | Gunter | Wrong design |
| First search | Study protocol for two randomized controlled trials examining the effectiveness and safety of current weekend allied health services and a new stakeholder-driven model for acute medical/surgical patients versus no weekend allied health services | Haines | Wrong outcome |
| First search | The Impact of Lean Management Implementation on Waiting Time and Satisfaction of Patients and Staff at an Outpatient Pharmacy of a Comprehensive Cancer Center in Jordan | Hammoudeh | Wrong design |
| First search | Application of case mix index in the allocation of nursing human resources | Han | Wrong design |
| First search | Leadership facilitation strategies to establish evidence-based practice in an acute care hospital | Hauck | Wrong/no intervention |
| First search | Do Technical Aids for Patient Handling Prevent Musculoskeletal Complaints in Health Care Workers?-A Systematic Review of Intervention Studies | Hegewald | Wrong publication type |
| First search | Evaluating a psychological support service focused on the needs of critical care and theatre staff in the first wave of COVID-19 | Herron | Wrong design |
| First search | Work-practice changes associated with an electronic emergency department whiteboard | Hertzum | Wrong/no intervention |
| Update | De-stressing From Distress Preliminary Evaluation of a Nurse-Led Brief Debriefing Program | Holbert | Wrong outcome |
| Update | Effect of safety and security equipment on patient and visitor violence towards nurses in multiple public hospitals of China during the COVID-19 pandemic: a retrospective, difference-in- difference analysis | Hu | Wrong/no intervention |
| First search | Effect of Balint group training on burnout and quality of work life among intensive care nurses: A randomized controlled trial | Huang | Wrong/no intervention |
| First search | A Randomized Controlled Trial of Balint Groups to Prevent Burnout Among Residents in China | Huang | Wrong/no intervention |
| First search | Pharmacists contribute to the improved efficiency of medical practices in the outpatient cancer chemotherapy clinic | Iihara | Wrong outcome |
| First search | The Huddling Program: effects on empowerment, organizational commitment and ego-resilience in clinical nurses - a randomized trial | Im | Wrong/no intervention |
| First search | Cognitive load in 3d and 2d minimally invasive colorectal surgery | Inama | Wrong/no intervention |
| First search | Wellness room as a strategy to reduce occupational stress: quasi-experimental study | Jacques | Wrong/no intervention |
| First search | Change in nurses' psychosocial characteristics pre- and post-electronic medical record system implementation coinciding with the SARS-CoV-2 pandemic: pre- and post-cross-sectional surveys | Jedwab | Wrong/no intervention |
| First search | Doctor of the Week: Effects on Billing, Time to Surgery, and Education | Jensen | Wrong publication type |
| First search | Benefit of a nurse-led telephone-based intervention prior to the first urogynecology outpatient visit: a randomized-controlled trial | Jimenez Torres | Wrong publication type |
| First search | Effectiveness Of Participatory Soft Skills Training On Quality Of Care, Clinical Communication Skills, Job Satisfaction And Self-Efficacy Among Nurses Working In Tertiary Care Hospitals: A Quasi-Experimental Study | Khan | Wrong design |
| Update | The Effect of Nurse Support Programs on Job Satisfaction and Organizational Behaviors among Hospital Nurses: A Meta-Analysis | Kim | Wrong publication type |
| First search | Effectiveness of team nursing compared with total patient care on staff wellbeing when organizing nursing work in acute care wards: a systematic review | King | Wrong publication type |
| First search | Randomized Trial of Therapy Dogs Versus Deliberative Coloring (Art Therapy) to Reduce Stress in Emergency Medicine Providers | Kline | Wrong/no intervention |
| First search | Implementation and Assessment of Mentoring and Professionalism in Training (MAP-IT): A Humanistic Curriculum as a Tool to Address Burnout in Surgical Residents | Kobritz | Wrong/no intervention |
| First search | Impact of Attending Physicians' Comments on Residents' Workloads in the Emergency Department: Results from Two Japan Randomized Controlled Trials | Kuriyama | Wrong outcome |
| First search | Effect on Patient Safety of a Resident Physician Schedule without 24-Hour Shifts | L and rigan | Wrong outcome |
| First search | Perceived effects of the 16-hour workday restriction on surgical specialties: Quebec's experience | Lachance | Wrong design |
| First search | No-sedation during mechanical ventilation: impact on patient's consciousness, nursing workload and costs | Laerkner | Wrong/no intervention |
| Update | Application effect of multidisciplinary collaborative nursing process in emergency care of patients with hypertensive cerebral hemorrhage | Li | Wrong outcome |
| First search | Protocol for a randomized trial of an interprofessional team-delivered intervention to support surrogate decision-makers in ICUs | Lincoln | Wrong publication type |
| First search | The Effect of a Freely Available Flipped Classroom Course on Health Care Worker Patient Safety Culture: A Prospective Controlled Study | Ling | Wrong/no intervention |
| First search | Conducting a representative national randomized control trial of tailored clinical decision support for nurses remotely: Methods and implications | Lopez | Wrong outcome |
| Update | Fostering positive emotions, psychological well-being, and productive relationships in the intensive care unit: A before-and-after study | Lovell | Wrong/no intervention |
| Update | Implementing workplace health promotion in nursing – A process evaluation in different care settings | Lützerath | Wrong outcome |
| First search | Canine-Assisted Therapy Improves Well-Being in Nurses | Machova | Wrong outcome |
| First search | A multicenter, randomized controlled, non-inferiority trial, comparing nasal high flow with nasal continuous positive airway pressure as primary support for newborn infants with early respiratory distress born in Australian non-tertiary special care nurseries (the HUNTER trial): study protocol | Manley | Wrong design |
| Update | The effect of balint practice on reducing stress, anxiety and depression levels of psychiatric nurses and improving empathy level | Mao | Wrong/no intervention |
| Update | Randomized trial of personalized psychological feedback from a longitudinal online survey and simultaneous evaluation of randomized stepped wedge availability of in-person peer support for hospital staff during the COVID-19 pandemic | Maunder | Wrong/no intervention |
| Update | Testing an Intervention to Improve Health Care Worker Well-Being During the COVID-19 Pandemic A Cluster Randomized Clinical Trial | Meredith | Wrong/no intervention |
| First search | Blood glucose control using an artificial pancreas reduces the workload of ICU nurses | Mibu | Wrong/no intervention |
| First search | Cluster-randomized trial evaluating a complex intervention to improve mental health and well-being of employees working in hospital - a protocol for the SEEGEN trial | Mulfinger | Wrong publication type |
| Update | Impact of transformational leadership intervention of head nurses among front-line soldiers (staff nurses) working in government hospital | Mushtaq | No full-tekst |
| First search | Design of the DISCovery project: tailored work-oriented interventions to improve employee health, well-being, and performance-related outcomes in hospital care | Niks | Wrong publication type |
| First search | Protecting and promoting mental health of nurses in the hospital setting: Is it cost-effective from an employer's perspective | Noben | Wrong/no intervention |
| First search | Comparative cost-effectiveness of two interventions to promote work functioning by targeting mental health complaints among nurses: pragmatic cluster randomized trial | Noben | Wrong/no intervention |
| Update | Comparison of two debriefing methods for nurse orientation | Persico | Wrong/no intervention |
| First search | Impact of Medical Scribes on Physician and Patient Satisfaction in Primary Care | Pozdnyakova | Wrong population |
| First search | Attentional Failures Are Correlated with Serious Medical Errors in Resident Physicians | Rahman | Wrong publication type |
| First search | Promoting Well-being Among Neurology Residents: A Data-Driven Approach | Ramanan | Wrong outcome |
| First search | Registered nurses job demands in relation to sitter use: A nested case-control study | Rochefort | Wrong design |
| First search | Smart agent system for insulin infusion protocol management: a simulation-based human factors evaluation study | Rosen | Wrong/no intervention |
| Update | Virtual Scribes and Physician Time Spent on Electronic Health Records | Rotenstein | Wrong outcome |
| First search | Staff satisfaction between 2 models of care for the chronically critically ill | Roulin | Wrong outcome |
| Update | Final year medical students as Assistants in Medicine in the emergency department: A pilot study | Rupasinghe | Wrong design |
| First search | Holding hands: Making a difference to junior doctor wellbeing | Sanwo | Wrong publication type |
| First search | Evaluation of Staff Satisfaction After Implementation of a Surgical Safety Checklist in the Ambulatory of an Oral and Maxillofacial Surgery Department and its Impact on Patient Safety | Schmitt | Wrong population |
| First search | Implementation of a multiprofessional, multicomponent delirium management guideline in two intensive care units, and its effect on patient outcomes and nurse workload: a pre-post design retrospective cohort study | Schubert | Wrong outcome |
| Update | Structured interdisciplinary bedside rounds improve interprofessional communication and workplace efficiency among residents and nurses on an inpatient internal medicine unit | Schwartz | Wrong/no intervention |
| First search | Continuous subcutaneous glucose monitoring at the intensive care unit: nursing workload reduction and cost benefit analysis | Sechterberger | Wrong publication type |
| First search | The effect of introducing an on-call system for attending physicians | Sekoguchi | No full-text in English |
| First search | A workplace organizational intervention to improve hospital nurses' and physicians' mental health: study protocol for the Magnet4Europe wait list cluster randomized controlled trial | Sermeus | Wrong publication type |
| First search | Bubble CPAP and oxygen for child pneumonia care in Malawi: a CPAP IMPACT time motion study | Sessions | Wrong outcome |
| First search | Navigating the storm: structuring and supporting junior doctor wellbeing during the COVID-19 pandemic | Shahi | Wrong publication type |
| First search | Comparative Study of Job Burnout Among Critical Care Nurses With Fixed and Rotating Shift Schedules | Shamali | Wrong design |
| Update | Positive effect of Balint group on burnout and self-efficacy of head nurses in China: a randomized controlled trial | Shan | Wrong/no intervention |
| First search | Nurses' perceptions and satisfaction with the use of insulin pen devices compared with insulin vial and syringes in an inpatient setting | Shogbon | Wrong design |
| First search | Physician burnout: can we make a difference together? | Siedsma | Wrong publication type |
| Update | Patient Safety Outcomes under Flexible and Standard Resident Duty-Hour Rules | Silber | Wrong/no intervention |
| First search | Nurse-patient assignment models considering patient acuity metrics and nurses' perceived workload | Sir | Wrong design |
| First search | A strategy for expanding infection prevention resources to support organizational growth | Smathers | Wrong/no intervention |
| First search | Introducing an integrated intermediate care unit improves ICU utilization: a prospective intervention study | Solberg | Wrong outcome |
| First search | Neuroradiologist Coverage Improves Resident Perception of Educational Experience, Referring Physician Satisfaction, and Turnaround Time | Spitler | Wrong design |
| First search | Reducing waste and improving patient safety: Introduction of the on-call doctor's bag | Stahl | Wrong publication type |
| First search | The Effect of Caring Training on the Implementation of Caring Behavior and Work Culture of Nurses in Providing Services to COVID-19 Patients in an Indonesian National Referral Hospital | Susanti | Wrong/no intervention |
| First search | Environmental psychology effects on mental health job satisfaction and personal wellbeing of nurses | Tavakkoli | Wrong design |
| First search | Stimulating Employability and Job Crafting Behavior of Physicians: A Randomized Controlled Trial | van Leeuwen | Wrong/no intervention |
| Update | Stimulating Employability and Job Crafting Behavior of Physicians: A Randomized Controlled Trial | van Leeuwen | Wrong/no intervention |
| First search | An electrocardiogram technician improves in-hospital first medical contact-to-electrocardiogram times: a cluster randomized controlled interventional trial | van Tulder | Wrong outcome |
| First search | Health-promoting work schedules: protocol for a large-scale cluster randomized controlled trial on the effects of a work schedule without quick returns on sickness absence among healthcare workers | Vedaa | Wrong publication type |
| Update | Reading room assistants to reduce workload and interruptions of radiology residents during on-call hours: Initial evaluation | Velleman | Wrong design |
| First search | The Impact of an Integrated Electronic Health Record Adoption on Nursing Care Quality | Walker-Czyz | Wrong publication type |
| First search | Active Intervention Can Decrease Burnout In Ed Nurses | Wei | Wrong/no intervention |
| First search | Managing Acute Behavioral Disturbances in the Emergency Department Using the Environment, Policies and Practices: A Systematic Review | Weil | Wrong publication type |
| First search | Colleagues Meeting to Promote and Sustain Satisfaction (COMPASS) Groups for Physician Well-Being: A Randomized Clinical Trial | West | Wrong/no intervention |
| First search | Effects of group psychological counselling on transition shock in newly graduated nurses: A quasi-experimental study | Xu | Wrong/no intervention |
| Update | Learning from a cluster randomized controlled trial to improve healthcare workers’ access to prevention and care for tuberculosis and HIV in Free State, South Africa: the pivotal role of information systems | Yassi | Wrong/no intervention |
| First search | Waterfalls and Handoffs: A Novel Physician Staffing Model to Decrease Handoffs in a Pediatric Emergency Department | Yoshida | Wrong design |
| First search | A Study on the Improvement of Nursing Interruption Risk by a Closed-Loop Management Model | Zhang | Wrong/no intervention |
| First search | Modifying head nurse messages during daily conversations as leverage for safety climate improvement: a randomized field experiment | Zohar | Wrong outcome |
